# ROCK2 Confers Acquired Gemcitabine Resistance in Pancreatic Cancer Cells by Upregulating Transcription Factor ZEB1

**DOI:** 10.3390/cancers11121881

**Published:** 2019-11-27

**Authors:** Yang Zhou, Yunjiang Zhou, Keke Wang, Tao Li, Minda Zhang, Yunjia Yang, Rui Wang, Rong Hu

**Affiliations:** 1 State Key Laboratory of Natural Medicines, School of Basic Medicine and Clinical Pharmacy, China Pharmaceutical University, Nanjing 210009, China; zyangcpu@163.com (Y.Z.); zyjcqmu@163.com (Y.Z.); wang21569532@163.com (K.W.); lt9868168@126.com (T.L.); dadagubao@163.com (M.Z.); cynthia_y9445@163.com (Y.Y.); cpu_wangrui@163.com (R.W.); 2 School of Basic Medicine and Clinical Pharmacy, China Pharmaceutical University, Nanjing 210009, China

**Keywords:** ROCK2, pancreatic cancer cells, gemcitabine, chemoresistance, ZEB1

## Abstract

Resistance to chemotherapy is a major clinical challenge in the treatment of pancreatic ductal adenocarcinoma (PDAC). Here, we provide evidence that Rho associated coiled-coil containing protein kinase 2 (ROCK2) maintains gemcitabine resistance in gemcitabine resistant pancreatic cancer cells (GR cells). Pharmacological inhibition or gene silencing of ROCK2 markedly sensitized GR cells to gemcitabine by suppressing the expression of zinc-finger-enhancer binding protein 1 (ZEB1). Mechanically, ROCK2-induced sp1 phosphorylation at Thr-453 enhanced the ability of sp1 binding to ZEB1 promoter regions in a p38-dependent manner. Moreover, transcriptional activation of ZEB1 facilitated GR cells to repair gemcitabine-mediated DNA damage via ATM/p-CHK1 signaling pathway. Our findings demonstrate the essential role of ROCK2 in EMT-induced gemcitabine resistance in pancreatic cancer cells and provide strong evidence for the clinical application of fasudil, a ROCK2 inhibitor, in gemcitabine-refractory PDAC.

## 1. Introduction

With a 5-year survival rate less than 5% [1], pancreatic ductal adenocarcinoma (PDAC) has the propensities of difficult diagnosis, early metastasis, and treatment-resistance [2]. As of yet, radical resection are not suitable for most patients, and chemotherapy remains the most frequent treatment option for advanced PDAC [3]. However, PDAC patients treated with chemotherapeutic drugs tend to develop chemotherapy resistance and ultimately lead to treatment failure [4,5]. Therefore, it is imperative to identify adjuvants that can improve the efficacy of chemotherapeutic agents. Gemcitabine, a deoxycytidine analogue, produces a wide range of anticancer activity in various epithelial cancers, such as bladder, non-small cell lung cancers, and PDAC [6,7]. Nevertheless, gemcitabine is not satisfactory as the first-line agent for pancreatic cancer due to the endogenous and exogenous drug resistance. The endogenous drug resistance mainly refers to the changes in drug metabolism, drug transport mechanism, and abnormal activation and inactivation of intracellular signaling pathways, while exogenous drug resistance is mainly caused by drug delivery hindrance [8,9]. 

Rho-associated coiled-coil kinases (ROCKs) as serine threonine kinases, are involved in actin cytoskeleton assembly, cell proliferation, cytokinesis, apoptosis, and migration [10]. As the members of the ROCKs family, ROCK1 and ROCK2 share 64% amino acid sequence homology and regulate different cellular functions. Recent studies have shown that ROCK2 is closely related to chemotherapy resistance. ROCKs inhibitors Y-27632 or fasudil treatment sensitize pancreatic cancer stem cells to gemcitabine by inhibiting ROCK kinase activity [11]. Pretreating pancreatic tumor with fasudil relax the surrounding tissues, and enhance the distribution of chemotherapeutic drugs in the Pdxl-Cre, LSL-KrasG12D/+, LSL-Trp53R172H/+ (KPC) mouse model [12]. We previously show that fasudil reverses drug resistance in temozolomide-resistant gliomas via inhibition of ROCK2/ABCG2 signaling pathway [13].

Epithelial mesenchymal transformation (EMT), a process of transforming adherent epithelial cells into fibroblasts, is found to be associated with drug resistance in pancreatic cancer [14,15,16]. Suppression of EMT increases the expression of nucleoside transporters and sensitizes gemcitabine treatment in mouse model [17]. Gemcitabine resistant pancreatic cancer cells have acquired EMT phenotype and highly express mesenchymal marker [18]. The activity of ROCK2 is found to be positively correlated with the migration and invasion in a variety of tumors such as glioblastoma [19], osteosarcoma [20], colorectal cancer [21], and other tumors. All of these highlight the role of ROCK2 as a driver for chemoresistance and an inducer of EMT. Nonetheless, the role of EMT in gemcitabine acquired resistance of pancreatic cancer cells and the involvement of ROCK2 in this process are still poorly understood.

In our current study, we focused on exploring the role of ROCK2 in EMT-elicited resistance to gemcitabine in gemcitabine resistant pancreatic cancer (GR) cells. Results showed that inhibition of ROCK2 increased the sensitivity of GR cells to gemcitabine in vitro and in vivo. Furthermore, ZEB1-mediated DNA damage repair played an essential role in ROCK2-conferred gemcitabine resistance. Mechanically, ROCK2 promoted gemcitabine resistance through p38/sp1/ZEB1 signaling pathway. Our data suggest that targeting the ROCK2 signaling pathway appears to be a promising approach against gemcitabine resistance in pancreatic cancer.

## 2. Methods

### 2.1. Cells Culture

SW1990 and gemcitabine-resistant SW1990 cell (SW1990/GEM) were kindly provided by Prof. Feng Qian (Tsinghua University, Beijing, China). Panc-1 cell was obtained from Cell Bank of the Chinese Academic of Sciences (Shanghai, China). Gemcitabine-resistant Panc-1 cell (Panc-1/GEM) was established as previously described [22]. All cell lines were maintained in DMEM medium (10% fetal bovine Serum, 100 U/mL of penicillin, and 100 mg/mL of streptomycin) and cultured in a humidified incubator with 5% CO_2_, at 37 °C. 

### 2.2. Reagents

Fasudil and SB203580 were purchased from Selleck Chemicals Inc. Gemcitabine, lysophosphatidic acid (LPA), Anisomycin, and Plicamycin (Mithramycin A) were purchased from Sigma-Aldrich (St. Louis, MO, USA). RIPA buffer solution, the Cell Cycle and Apoptosis Analysis Kit, nuclear and cytoplasmic protein extraction kit, Dual Luciferase Reporter Gene Assay Kit, and CHIP assay Kit were obtained from Beyotime Biotechnology (Hangzhou, China). Comet electrophoresis detection cell damage assay kit obtained from Keygen Biotec (shanghai, China). AceQ qPCR SYBR Green Master Mix was purchased from Vazyme (Nanjing, China), and First-strand cDNA Synthesis super Mix kit was from TransGen Biotech (Beijing, China). TRIzol reagent (Invitrogen; Carlsbad, CA, USA). BrdU assay kit was purchased from CST. Primary antibodies we used were shown as following: p-ROCK2 (Tyr-722), p-sp1 (Thr-T453), p-γ-H_2_AX (S139), cleaved parp1, and Twist were purchased from Abcam (Cambridge, MA, USA). Snail, Slug, ZEB1, Vimentin, E-cadherin, ATM, CHK1, p-CHK1, sp1, p38, and p-p38 (Thr180/Tyr182) were purchased from Cell Signaling Technology. ROCK1 and β-actin were purchased from Bioworld Technology Inc. (Minnesota, MN, USA). ZEB2 were purchased from Santa Cruz Biotechnology (Santa Cruz, CA, USA). Fibronectin was purchased from Proteintech Group Inc. P-ROCK1 (Thr455 + Ser456) was purchased from Bioss (Beijing, China). Lamin A antibody was purchased from Sigma-Aldrich (St. Louis, MO, USA).

### 2.3. Western Blotting Analysis

RIPA buffer solution and nuclear and cytoplasmic protein extraction kit were used for extracting the total protein and nucleus protein, respectively. Protein expression was detected using indicated primary antibody. All the experimental steps were performed in accordance with the standard manufacturer’s instructions.

### 2.4. RNA Extraction and qRT-PCR

Total RNA was extracted using the TRIzol reagent. The purity and concentration of the extracted RNA were measured at 280 and 260 nm. RNA samples were reverse transcribed to cDNA using the first-strand cDNA synthesis super Mix kit and subjected to quantitative PCR, which was performed using AceQ qPCR SYBR Green Master Mix with the Light-Cycler 96 Real-Time PCR System (Roche). The primer sequences used in this study are shown in Table 1.

### 2.5. Colony-Formation Assay 

Cells were seeded into six-well plate (500 cells/well) and incubated for 24 h. After specific treatment, cells were cultured in drug-free medium for another two weeks. Cells were stained with 0.1% crystal violet after fixing with 4% formaldehyde. The number of colonies was then counted macroscopically.

### 2.6. Brdu Incorporation Assay

Five thousand cells per well were implanted on 96 well plates. After specific treatment, cells were incubated with BrdU for two hours, and the absorbance of cell proliferation was determined according to the manufacturer’s instructions. 

### 2.7. Transfection ShRNA and Plasmid Constructs

The method of transfecting short-hairpin RNA (shRNA) was described as previously reported [22]. For transfection of shRNA, lentiviral particles shRNA encoding targeting genes, or scramble shRNA (Sigma, St. Louis, MO, USA) were used. Puromycin (5 μg/mL) was used to select transfected cells after transfected with indicated shRNA for three days. For transfection of plasmid, empty vector or ZEB1 plasmid was used (Shanghai Fubio Co., LTD, Shanghai, China). As for cells transfection, ExFect transfection reagent (Vazyme, Nanjing, China) was used according to the manufacturer’s instruction. The expression levels of indicated proteins were detected to verify transfected cells. 

### 2.8. Chromatin Immunoprecipitation

ChIP assay was performed using the ChIP assay kit following the instructions of the manufacturer. Briefly, cells were cross-linked and sonicated and then immunoprecipitation was performed. Cell lysates were incubated with anti-human sp1 antibody overnight at 4 °C, and IgG antibody was used as negative control. Then protein A/G beads were used for recovery of immunocomplexes. Purified immunoprecipitation DNA and input DNA were analyzed by semi-quantitative PCR. The primers used to amplify the genomic sequences of ZEB1 promoter fragments were listed in Table 2. 

### 2.9. Cytotoxicity Assay

Cytotoxicity of gemcitabine or fasudil in pancreatic cancer cells was determined using MTT assay. In brief, cells were seeded on a 96-well plate (5 × 10^3^ cells / well) and incubated for 24 h. After treatment with gemcitabine and fasudil alone or in combination for indicated time point, cells were added 20 μL of MTT per well (0.5 mg/mL) and incubated for another 4 h. An automated Microplated Reader Elx800 (Bio Tek Instruments Inc., Winooski, VT, USA) was used to determine the cell viability with absorbance at 570 nm. The inhibition rate was calculated using the formulation below: Inhibition rate = (1 − A_treated_/A_control_) × 100. Based on the Chou-Talalay combination index method, the combination index (CI) was calculated by Compusyn version 1.0 software (The ComboSyn, Inc., NJ, USA) [23].

### 2.10. Luciferase Assay

Cells were co-transfected with ZEB1 promoter luciferase constructs (Sangon, Shanghai, China) and pRL-TK Renilla plasmid (Promega). The pGL3 empty vector was used as a negative control. The lysate was prepared and the luciferase activity was detected using the dual luciferase assay kit according to the manufacturer’s protocol. The luciferase activity was normalized based on the Renilla activity. 

### 2.11. Comet Assay

Cells treated with different concentrations of gemcitabine at indicated time points were washed with cold PBS then centrifugally collected, and re-suspended with PBS. Subsequently, comet assay was performed according to the instructions of the manufacturer. Nuclear DNA and migrating DNA was observed using fluorescence microscope under excitation at 515–560 nm. The damage of DNA can be classified according to the ratio of comet tail DNA to total DNA.

### 2.12. Xenograft Model

Female nude mice (6-week old, Beijing Vitone River Laboratory Animal Technology Co., Ltd.) were randomly divided into eight experimental groups (six mice/group). Mice were subcutaneously injected with about 1.0 × 10^6^/mouse of SW1990/GEM cell, shCtrl-SW1990/GEM cell, ShROCK2-SW1990/GEM cell, and ShROCK2+ZEB1-SW1990/GEM cell, respectively. The mice were treated with gemcitabine or fasudil, when the tumor volume of mice reached about 100 mm^3^. In the treatment group, mice were intraperitoneally injected with gemcitabine (50 mg/kg, once every 4 days) or fasudil (20 mg/kg, once every 2 days) alone or in combination. At the same time, solvent was used as a blank control group. Mice tumor sizes were measured and recorded every 4 days during the treatment. Tumor volumes were calculated using formula: V = (1/2) a × b^2^, where “a” and “b” represent the tumor’s long axis and the short axis, respectively. Mice were killed after administration for 24 days, and the tumor xenografts were then resected and weighed. All experiments were performed in accordance with the National Institutes of Health Guide for the Care and Use of Laboratory Animals, and all animal experimental procedures were approved by Experimentation Ethics Review Committee of China Pharmaceutical (2019-09-001).

### 2.13. Immunohistochemistry

Immunohistochemical staining of indicated proteins was performed using immunohistochemistry kit (KeyGen, Nanjing, China) in accordance with the instructions of manufacturer. All the sections were photographed under an inverted fluorescence microscope (Nikon, Japan).

### 2.14. Statistical Analysis

All the results were obtained from at least three independent experiments and expressed as mean ± SD. Statistical analysis was performed with the *t*-test and one-way ANOVA (SPSS Software, Armonk, NY, USA) for two groups or multiple groups, respectively. Statistically significant difference was shown as * *p* < 0.05, ** *p* < 0.01, and *** *p* < 0.001.

## 3. Results

### 3.1. ROCK2 is Overexpressed in GR Cells, and Fasudil Plus Gemcitabine Synergistically Enhance the Sensitivity of GR Cells to Gemcitabine

**Figure 1:**
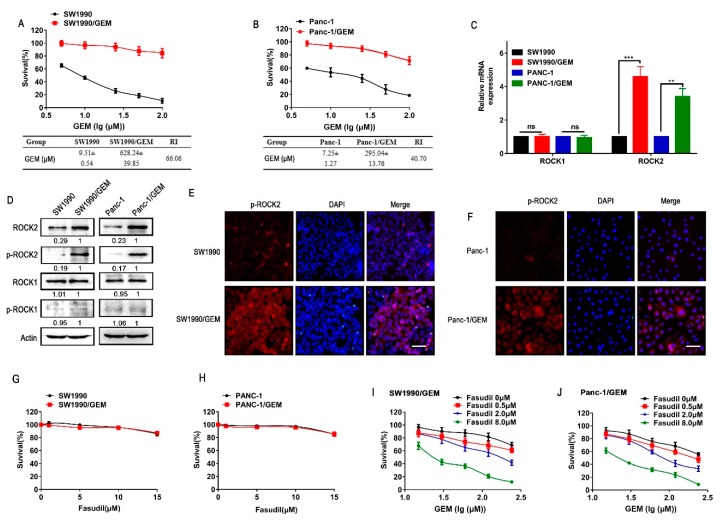
The synergistic effect of fasudil and gemcitabine on the growth of GR cells. (**A**,**B**) Increasing concentrations of gemcitabine were treated into gemcitabine resistant pancreatic cancer (GR) cells and parental cells for 24 h, cells survival rate was detected by the MTT method. The IC50 values and drug resistance index (RI) of gemcitabine were measured. (**C**) Relative mRNA levels of ROCK1 and ROCK2 in GR cells and parental cells were detected by real-time PCR. (**D**) Relative protein levels of ROCK1, p-ROCK1, ROCK2, and p-ROCK2 in GR cells and parental cells were detected by western blot. (**E**,**F**) Immunofluorescence staining of p-ROCK2 in GR cells and parental cells. Scale bar 50 μm. (**G**–**J**) Cell viability was determined by MTT assay. G, H GR cells, and parental cells were treated with various doses of fasudil for 24 h. (**I**,**J**) GR cells were treated with indicated concentrations of fasudil and gemcitabine either alone or in combination for 24 h. All data represents three independent experiments and is presented as mean ± SD (* *p* < 0.05, ** *p* < 0.01, and *** *p* < 0.001).

According to MTT assay, GR cells (SW1990/GEM and Panc-1/GEM) showed higher IC_50_ values compared with parental cells (SW1990 and Panc-1). The drug resistance index (RI) in SW1990/GEM and Panc-1/GEM were 66.06 and 40.70, respectively (Figure 1A,B). As shown in Figure 1C,D, the expression and phosphorylation of ROCK2 were significantly higher in GR cells than those in parental cells, while the expression and phosphorylation of ROCK1 were indistinguishable between GR cells and parental cells. Similarly, the immunocytochemistry assay further validated the upregulation of p-ROCK2 in GR cells (Figure 1E,F). According to the construction method of GR cells, we speculated that the upregulation of ROCK2 in GR cells might be due to gemcitabine-induced stress or gemcitabine selection in parental cells. However, gemcitabine treatment did not induce upregulation of ROCK2 in SW1990 and PANC-1 cells (Supplementary Figure S1a,b). This excludes that the overexpression of ROCK2 is caused by gemcitabine stress. In order to explore whether ROCK2 was upregulated under gemcitabine selection, we compared the ROCK2 expression in parental cells and selected parental cells, which could stably grow in the medium with 5.0 μm gemcitabine. ROCK2 was found upregulated in survived cells compared with untreated cells although there was no significant difference (Supplementary Figure S1c). We speculated that under stimulation of gemcitabine, cells with low expression of ROCK2 died, while cells with high expression or adaptive up-regulation of ROCK2 survived. In recent years, fasudil has been found to induce apoptosis in cancer cells [24,25]. Unexpectedly, fasudil treatment had no significant inhibitory effect on the growth of GR cells and parental cells (Figure 1G,H). In the meantime, non-lethal dose of fasudil treatment sensitized GR cells to gemcitabine as demonstrated by the decreased IC_50_ values of gemcitabine (Figure 1I,J and Table 3). CI values were calculated to reflect the synergistic effect of fasudil and gemcitabine (Table 4). However, fasudil and gemcitabine had a weak synergistic effect or only an additive effect on parental cells (Supplementary Figure S2). It might be due to that the low p-ROCK2 expression of parental cells or the high sensitivity of parental cells to gemcitabine masked the effect of fasudil. Moreover, fasudil was also synergistic with other drugs such as 5FU, paclitaxel, and cisplatin in GR cells (Supplementary Figure S3a–c). These demonstrated that targeting ROCK2 might be a potential strategy to improve the efficacy of various anticancer drugs in the treatment of refractory pancreatic cancer. 

### 3.2. Pharmacological Inhibition of ROCK2 Renders DNA Damage Induced by Gemcitabine

**Figure 2:**
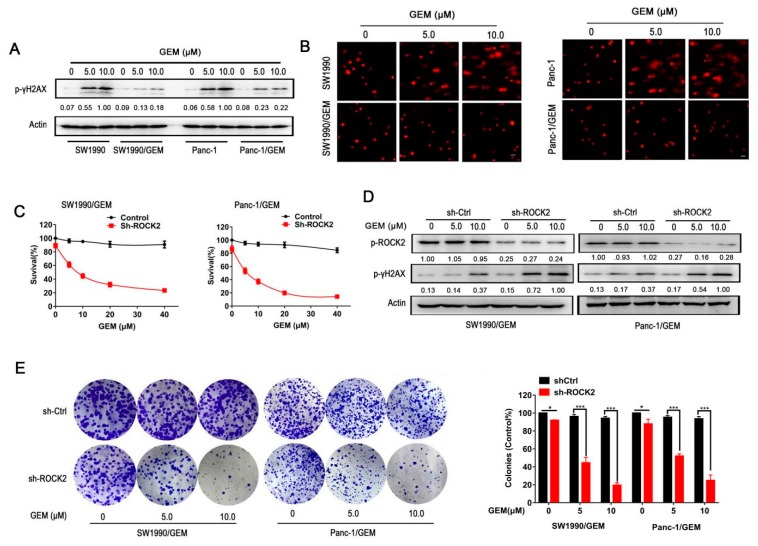
Knockdown of ROCK2 sensitizes GR cells to gemcitabine by inducing DNA damage toxicity. Parental cells or GR cells were treated with gemcitabine (5 μM and 10 μM) for 24 h. (**A**) Protein level of p-γH2AX was detected to reflect DNA damage induced by gemcitabine in pancreatic cancer cells. (**B**) Comet assay was performed to reflect the gemcitabine-induced DNA strand breaks. Scale bar 50 μm. (**C**) GR cells transfected with scramble or ROCK2-targeting short-hairpin RNA (shRNA), and then exposed to increasing concentrations of gemcitabine for 24 h. Cell viability was determined by MTT assay. (**D**) GR cells transfected with control shRNA (shCtrl-GR cells) and shROCK2 (shROCK2-GR cells) were treated with indicated concentrations of gemcitabine for 24 h, and then protein levels of p-ROCK2 and p-γH2AX was detected. (**E**) Mock and shROCK2-GR cells treated with gemcitabine (5 μM, 10 μM) were subjected to colony formation assay. Data represents three independent experiments and is presented as mean ± SD (* *p* < 0.05, ** *p* < 0.01, and *** *p* < 0.001).

Phosphorylation of γH_2_AX at S139 (p-γH_2_AX), a marker of DNA damage [26], was detected to show the DNA damage in GR cells and parental cells in response to gemcitabine treatment. As shown in Figure 2A, p-γH_2_AX was significantly upregulated in parental cells when treated with gemcitabine, but only slightly increased in GR cells. Consistently, the comet assay further confirmed the resistance of GR cells to gemcitabine-induced DNA damage (Figure 2B).

As shown in Supplementary Figure S4a,b, fasudil but not gemcitabine inhibited the phosphorylation of ROCK2 in a dose-dependent manner, indicating that activation of ROCK2 may help in maintaining the gemcitabine resistance. In order to test this, short-hairpin RNA (shRNA) targeting ROCK2 was transfected into GR cells, and the knockdown efficiency were detected in Supplementary Figure S5. As shown in Figure 2C, ablation of ROCK2 significantly sensitized GR cells to gemcitabine, which was consistent with the results in Figure 1I,J. In addition, knockdown of ROCK2 significantly enhanced gemcitabine-induced DNA damage in GR cells (Figure 2D). With the clonogenic assay, we found that ablation of ROCK2 resulted in the formation of fewer colonies in response to gemcitabine treatment in GR cells (Figure 2E). Furthermore, gemcitabine significantly reduced BrdU incorporation in shROCK2 GR cells in a dose-dependent manner (Supplementary Figure S6). All of these results indicate the important role of ROCK2 in gemcitabine resistance.

### 3.3. Inhibition of ROCK2 Partially Reverses EMT in GR Cells

**Figure 3:**
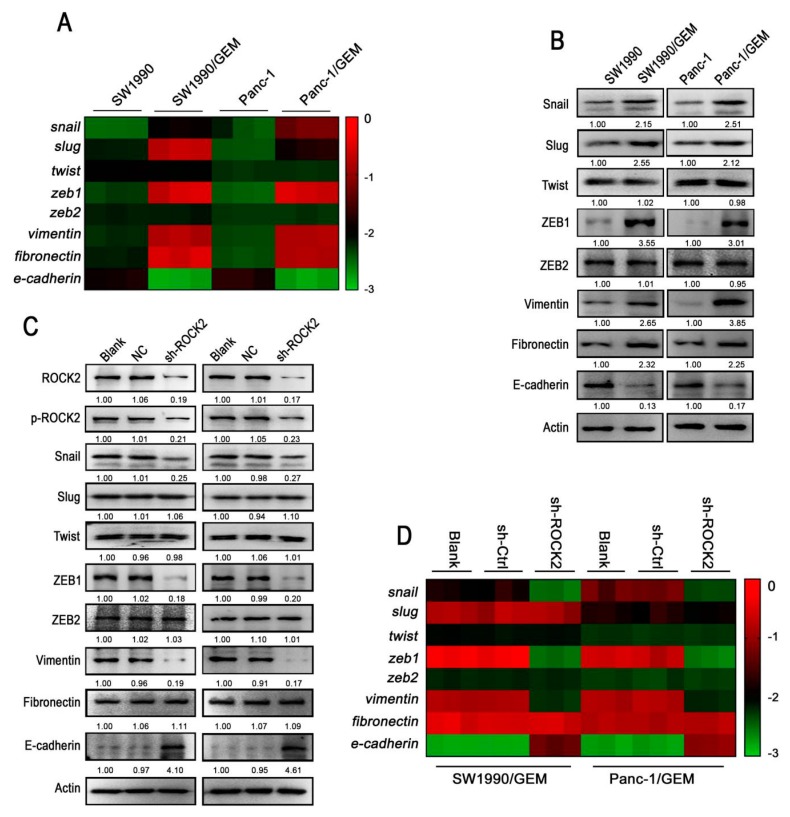
GR cells express higher level of mesenchymal marker than parental cells and inhibition of ROCK2 partially reverses EMT in GR cells. Relative mRNA and protein levels of mesenchymal markers and E-cadherin in GR cells and parental cells were detected by (**A**) real-time PCR and (**B**) western blot, respectively. The color of the heatmap represents value of −△Ct (means Ct _internal reference gene_ − Ct _the target gene_). Relative protein and mRNA levels of EMT markers in GR cells were detected by (**C**) western blot and (**D**) real-time PCR upon transfection with control shRNA or shROCK2. The color of heatmap represents value of −△Ct (means Ct _internal reference gene_ − Ct _the target gene_). Data represents three independent experiments.

As EMT phenotype of chemoresistant cancer cells has been documented [17,27], it is not surprising that the mRNA and protein expression levels of Snail, Slug, ZEB1, Vimentin, and Fibronectin were higher in GR cells compared with parental cells (Figure 3A,B). However, no significant difference in the expression of Twist and ZEB2 were observed between GR cells and parental cells. In addition, the lower expression level of E-cadherin in GR cells further confirmed the EMT phenotype of GR cells (Figure 3A,B). Although ROCK2 is considered to be involved in the EMT process, the exact regulatory mechanism has not been clearly elucidated. In order to determine whether the expression of ROCK2 was regulated by EMT, TGF-β1 was used to induced EMT in parental cells. TGF-β1 significantly increased the mRNA expression of mesenchymal markers and decreased the mRNA expression of E-cadherin (Supplementary Figure S7a,b). However, the protein and mRNA expression of ROCK2 was not changed significantly, indicating that ROCK2 expression was not affected by EMT. We found that pharmacological inhibition or RNA interference of ROCK2 resulted in the downregulation of Snail, ZEB1, Vimentin, and the upregulation of E-cadherin in GR cells (Supplementary Figure S8 and Figure 3C,D). However, ROCK2 blockade had no obvious effect on the expression of Slug, Twist, ZEB2, and Fibronectin. Thus, the expression of EMT markers was partially regulated by ROCK2 in pancreatic cancer cells. 

### 3.4. ZEB1 is the Dominant Factor for ROCK2-Mediated Gemcitabine Resistance in GR Cells

**Figure 4:**
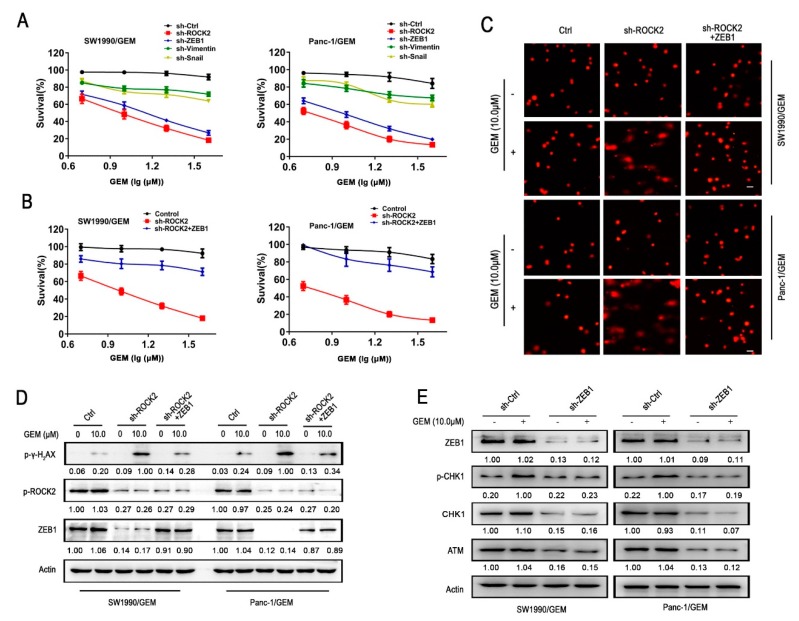
Knockdown of ROCK2 sensitizes GR cells to gemcitabine, which is prevented by re-expression of ZEB1. (**A**,**B**) Cells were treated with gemcitabine for 24 h, and cell viability was determined by MTT assay. (**A**) Scramble shRNA, shROCK2, shZEB1, shVimentin, and shSnail were transfected into GR cells respectively. (**B**) ZEB1-expressing plasmid (4 μg) was stably transfected into shROCK2-GR cells. (**C**,**D**) GR cells, shROCK2-GR cells, and shROCK2+ZEB1-GR cells were treated with or without gemcitabine (10 μM) for 24 h. (**C**) Ectopic expression of ZEB1 rescued DNA damage effect of gemcitabine in shROCK2-GR cells as performed by comet assay. Scale bar 50 μm. (**D**) Relative expression levels of indicated protein were detected by western blot. (**E**) GR cells transfected with control shRNA or sh-ZEB1 treated with or without gemcitabine (10 μM) for 24 h, and the indicated protein was detected by western blot. Data represents three independent experiments and is presented as mean ± SD (* *p* < 0.05, ** *p* < 0.01, and *** *p* < 0.001).

To investigate whether upregulation of ZEB1, Snail, and Vimentin induced by ROCK2 were responsible for the resistance of GR cells to gemcitabine, shRNA targeting Snail, Vimentin, and ZEB1 were transfected into GR cells, respectively. Of note, ZEB1-deletion significantly attenuated the resistance of GR cells to gemcitabine, which was similar to that of ROCK2-deletion, while silencing of Snail or Vimentin only slightly sensitized GR cells to gemcitabine (Figure 4A). As shown in Supplementary Figure S9, ZEB1 plasmid restored the reduction of ZEB1 expression caused by ROCK2 knockdown in a dose-dependent manner. Furthermore, re-expression of ZEB1 with overexpressing plasmid reversed ROCK2 knockdown-mediated sensitization of GR cells to gemcitabine (Figure 4B). Similarly, re-expression of ZEB1 suppressed ROCK2 knockdown-induced elevation of DNA damage in response to gemcitabine treatment in GR cells (Figure 4C,D). As shown in Figure 4E, ZEB1 blockade attenuated gemcitabine-induced activation of ATM/p-CHK1 signaling in GR cells. Furthermore, re-expression of ZEB1 prevented gemcitabine-induced apoptosis in GR cells transfected with shROCK2 (Supplementary Figure S10). In addition, overexpression of ZEB1 also significantly reversed ROCK2 ablation-mediated sensitivity of GR cells to 5-FU, paclitaxel and cisplatin (Supplementary Figure S11a–e and Table 5). Therefore, ZEB1-induced DNA damage repair is necessary for ROCK2-mediated resistance of GR cells to gemcitabine and other chemotherapeutics. 

### 3.5. ROCK2 Upregulates ZEB1 via the p38/sp1 Signaling Pathway but not Snail

Studies have shown that expression of some mesenchymal proteins, including ZEB1 can be regulated by snail in some cases [28]. The high level of Snail in GR cells prompted us to speculate the potential role of Snail in the regulation of ZEB1. However, knockdown of snail in GR cells did not cause obvious change in ZEB1 expression (Supplementary Figure S12a). Furthermore, the role of snail in ROCK2-mediated EMT was also addressed in parental cells. LPA is found to effectively induce the activation of RhoA/ROCKs [29]. As shown in Supplementary Figure S12b, p-ROCK2, Snail, and ZEB1 expression were obviously increased after stimulation of LPA in parental cells. However, knockdown of Snail had no obvious effect on LPA-mediated ZEB1 expression in parental cells.

**Figure 5:**
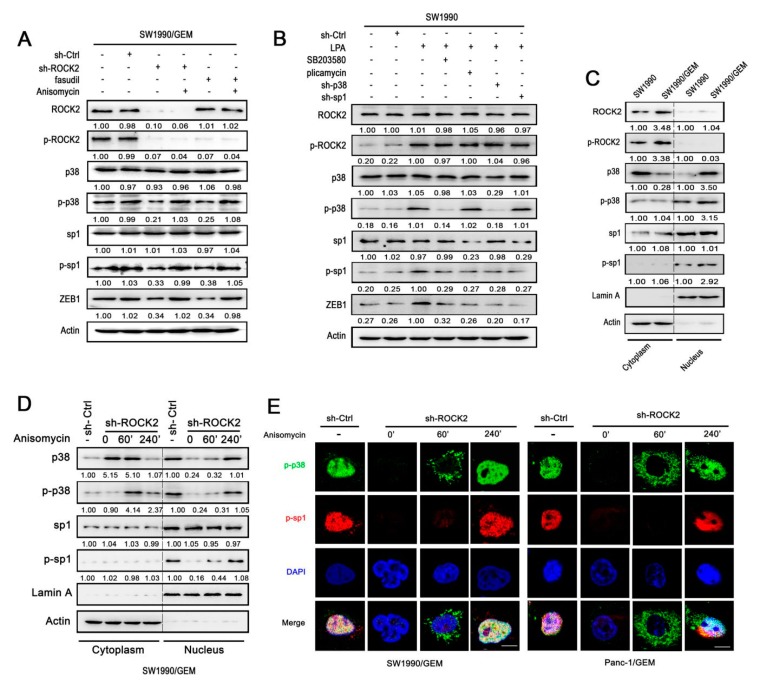
ROCK2 upregulates ZEB1 expression via p38/sp1 signaling pathway. (**A**) ShROCK2-SW1990/GEM cell treated with or without Anisomycin. SW1990/GEM treated with fasudil or Anisomycin alone or in combination. (**B**) SW1990 cell was pretreated with LPA, and then treated with SB203580, plicamycin, respectively. ShCtrl, Sh-p38, or sh-sp1 were transfected into SW1990 cell after pretreated with LPA, respectively. (**C**,**D**) Detection of the protein expression levels of p-ROCK2, p38, p-p38, sp1, and p-sp1 in nucleus and cytoplasm by western blot. (**C**) Differential expression of indicated proteins between SW1990/GEM and SW1990 in nucleus and cytoplasm. (**D**) Scramble shRNA or shROCK2 were transfected into SW1990/GEM. ShROCK2-SW1990/GEM was treated with Anisomycin for indicated time points. (**E**) Mock and shROCK2-SW1990/GEM cells were treated with or without anisomycin for 60 min and 240 min. Location and expression levels of p38, p-p38, sp1, and p-sp1 were analyzed by immunofluorescent staining. Scale bar 5 μm. Data represents three independent experiments.

To explore the potential mechanism of ROCK2 in enhancing ZEB1 expression, effects of ROCK2 on the p38 and sp1 signaling pathways were studied, since both sp1 and p38 are reported closely associated with EMT in many cancers [30,31]. GR cells expressed higher levels of p-p38 and p-sp1 compared with parental cells (Supplementary Figure S13a). Furthermore, pharmacological inhibition or silencing of ROCK2 induced reduction of p-ROCK2, p-p38, p-sp1, and ZEB1 in GR cells. However, Anisomycin (an activator of p38) treatment prevented the reduction of these proteins, except for p-ROCK2 (Figure 5A and Supplementary Figure S13b). It indicates that p38 and sp1 are downstream effectors of ROCK2, and ZEB1 is positively regulated by p38/sp1 signaling pathway. Further study showed that pharmacological inhibition and RNA interference of p38 or sp1 attenuated LPA-mediated upregulation of ZEB1 in parental cells (Figure 5B and Supplementary Figure S13c). In addition, protein level of p-p38 remained unchanged upon sp1 inhibition. Overall these suggest that the activation of p38/sp1 signaling is necessary for ROCK2-induced ZEB1 expression. 

### 3.6. ROCK2 Promotes p38 Nuclear Translocation and Activation of p38/sp1 Signaling Pathway

It has been reported that phosphorylation of sp1 facilities sp1-mediated transcription of target genes in the nucleus [32]. However, it remained unclear how ROCK2 induced activation of p38/sp1 signaling pathway in GR cells. Therefore, intracellular distribution of p-ROCK2, p38, p-p38, sp1, and p-sp1 were analyzed to explore their intrinsic relationship. GR cells expressed higher protein levels of p38, p-p38, and p-sp1 in the nucleus compared with parental cells (Figure 5C and Supplementary Figure S13d). However, ROCK2 and p-ROCK2 expression were mainly detected in the cytoplasm and were higher in GR cells compared with parental cells. In addition, sp1 was mainly detected in nucleus, but there was no difference between GR cells and parental cells. It suggests that the higher expression level of nuclear p-p38 in GR cells is probably responsible for the upregulation of p-sp1. Interestingly, ablation of ROCK2 enhanced the translocation of p38 from the nucleus to cytoplasm in GR cells. What is more, knockdown of ROCK2 induced reduction of p-p38 and p-sp1 in the nucleus (Figure 5D and Supplementary Figure S13e). Based on these findings above, we speculated that the accumulation of p-p38 in nucleus is probably responsible for ROCK2-mediated activation of sp1. As expected, activation of p38 with Anisomycin counteracted ROCK2 deletion-induced reduction and nuclear translocation of p-p38 in a time-dependent manner (Figure 5D and Supplementary Figure S13e). Moreover, p-sp1 was also upregulated in the nucleus in response to activation of p38. Furthermore, the immunostaining intensity of nuclear p-p38 and p-sp1 was significantly decreased after knockdown of ROCK2 (Figure 5E). However, after treated with Anisomycin for 60 min, p-p38 was reincreased in the cytoplasm, while p-sp1 was not changed obviously in the nucleus. Interestingly, p-p38 was redistributed from cytoplasm to nucleus and accompanied by the upregulation of p-sp1 in the nucleus after treated with Anisomycin for 240 min. Therefore, ROCK2-induced phosphorylation of p38 and p-p38 nuclear translocation are responsible for sp1 activation.

### 3.7. ROCK2 Enhances the Ability of sp1 Binding to the Promoter of ZEB1

**Figure 6:**
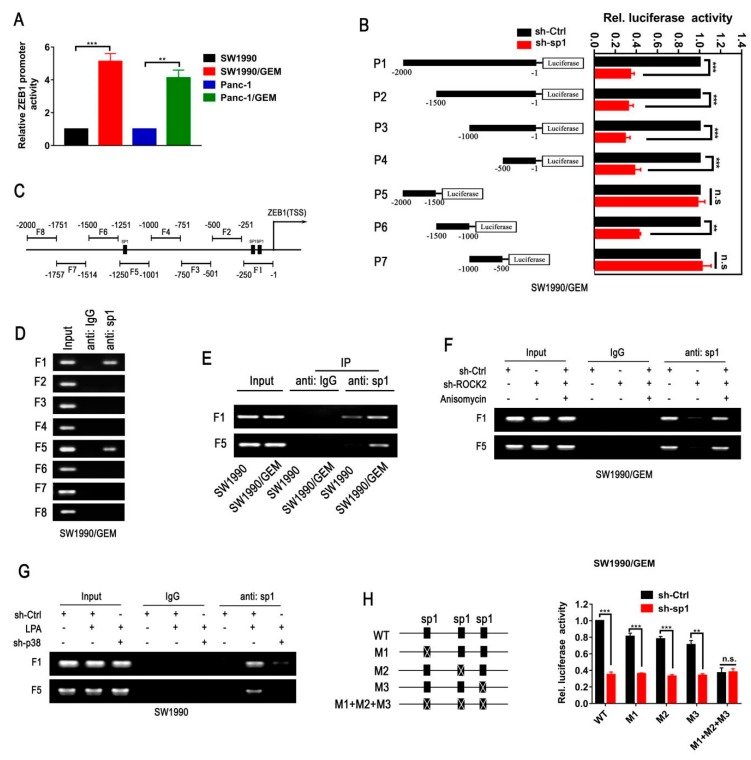
ROCK2 mediates ZEB1 transcription by enhancing sp1 recruitment on the ZEB1 promoter. (**A**–**C**) The dual luciferase reporter assay was used to analyze activation of the ZEB1 promoter. The luciferase activity was normalized based on the Renilla activity. ZEB1 promoter luciferase construct (nt −1 to nt −2000) was transiently transfected into (**A**) GR cells and parental cells. (**B**) Full-size and progressively deleted ZEB1 promoter luciferase constructs were transiently transfected into SW1990/GEM cell and shsp1-SW1990/GEM cell. pGL3 empty vector was used as a negative control and pRL-TK Renilla plasmid was used as an internal control. (**C**) The schematic diagram of PCR-amplified fragments of ZEB1 promoter. (**D**–**G**) The chromatin IP method was performed to detect the physical binding between sp1 and ZEB1 promoter regions. (**D,E**) The sp1 binding sites in ZEB1 promoter were detected by sp1 antibody in SW1990/GEM cell and SW1990 cell. Immunoglobulin G (IgG) was used as a negative control. (**F**) Anisomycin was treated into SW1990/GEM cell after transfected with ShROCK2. (**G**) SW1990 cell was transfected with scramble shRNA or p38-targeting shRNA after treated with LPA. (**H**) Luciferase activity of the ZEB1 promoter constructs with wild type or mutated binding sites for sp1 were detected. Representative statistics derived from three independent experiments are expressed as means ± SD (* *p* < 0.05, ** *p* < 0.01, and *** *p* < 0.001).

To investigate the effect of sp1 on the transcription of ZEB1, qPCR analysis was performed. We found that ZEB1 expression was reduced by a knockdown of sp1 in GR cells (Supplementary Figure S14a). A luciferase reporter assay was performed to further explore the mechanism of sp1-mediated ZEB1 transcription. The higher luciferase activity of ZEB1 promoter was observed in GR cells compared with parental cells (Figure 6A). Moreover, the luciferase activity of ZEB1 promoter was attenuated by sp1 knockdown in GR cells (Figure 6B and Supplementary Figure S14b). These results above further confirm that sp1 is necessary for ZEB1 expression in GR cells. To identify the potential binding regions of sp1 on the ZEB1 promoter, full-size, and deletion mutation of ZEB1 promoter luciferase constructs were transiently transfected into GR cells. Sp1 knockdown significantly decreased the relative luciferase activity of ZEB1 promoter fragments, except for P5 and P7, indicating that the interaction of sp1 with P4 and P6 regions are involved in ZEB1 transcription.

The potential sp1 binding sites on ZEB1 promoter was identified within F1 and F5 regions using Jaspar database (http:/jaspar.genereg.net/; Figure 6C). ChIP analysis roughly observed that sp1 only bound to the F1 and F5 regions on ZEB1 promoter, which covered the predicted binding sites (Figure 6D and Supplementary Figure S14c). Furthermore, the binding efficiency of sp1 to F1 and F5 regions was stronger in GR compared with parental cells (Figure 6E and Supplementary Figure S14d). CHIP assay was conducted to verify the functional significance of ROCK2/p38 signaling in this process. Knockdown of ROCK2 reduced the binding efficiency of sp1 to F1 and F5 regions, which was prevented by Anisomycin treatment (Figure 6F and Supplementary Figure S14e). Similarly, LPA treatment-mediated enhanced binding efficiency of sp1 to F1 and F5 regions in parental cells was reversed by knockdown of p38 (Figure 6G and Supplementary Figure S14f). To validate whether the three potential binding sites were reasonable for the transcriptional activation of ZEB1 promoter, mutations of single site (Mut1, Mut2, and Mut3) and in combination (Mut1–3) were performed. As expected, mutation of single site resulted in a reduction of promoter activity, which was further reduced by mutation of all the three sites (Figure 6H and Supplementary Figure S14g). In addition, deletion of sp1 reduced the luciferase activity of wild type and single site mutation constructs. In contrast, knockdown of sp1 exhibited no effect on the luciferase activity of the Mut1-3 construct. It suggests that all of these three potential binding sites are necessary for sp1-mediated ZEB1 transcription. Collectively, ROCK2 induces the upregulation of ZEB1 by enhancing the ability of sp1 binding to the ZEB1 promoter in a p38-dependent manner.

### 3.8. ROCK2 Promotes Gemcitabine Resistance in Nude Mice Dependent on ZEB1

**Figure 7:**
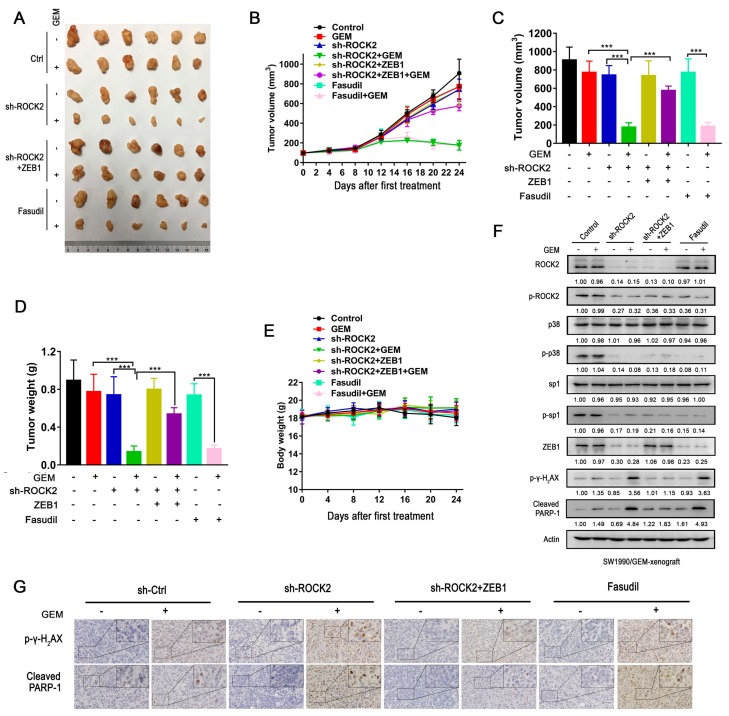
Gemcitabine’s effect on the SW1990/GEM cell-derived xenograft tumor is enhanced by inhibition of ROCK2, but is attenuated by overexpression of ZEB1. The tumor-bearing mice were intraperitoneally injected with gemcitabine (50 mg/kg, every four days), fasudil (20 mg/kg, every two days), or solvent for 24 days. (**A**) Tumors were obtained from the nude mice at the end of the experiment. (**B**,**C**) The tumor size in each group was measured by Vernier caliper every four days. (**D**) Tumor weight and (**E**) body weight. Relative expression of indicated proteins in tumor tissues were detected by (**F**) western blot and (**G**) IHC. All images were shown at ×400. The results of histogram were expressed as mean ± SD, *n* = 6 (*p < 0.05, ** *p* < 0.01, and *** *p* <0.01).

The xenograft model was used to determine the effect of ROCK2/ZEB1 signaling pathway on gemcitabine resistance in vivo. We found that gemcitabine only slightly inhibited tumor growth of the SW1990/GEM cell, whereas significantly reduced the volume and weight of the SW1990/GEM-shROCK2 cell-driven tumor (Figure 7A–D). In contrast, overexpression of ZEB1 prevented gemcitabine-induced inhibition of tumor growth of the SW1990/GEM-shROCK2 cell. This further confirmed the ZEB1-dependent gemcitabine resistance of the SW1990/GEM cell mediated by ROCK2 in vivo. Fasudil combined with gemcitabine led to a synergistic inhibitory effect on the growth of SW1990/GEM cell-driven xenograft (Figure 7A–D). The unchanged body weight of nude mice in combination group proved the safety of fasudil combined with gemcitabine in vivo (Figure 7E). Both western blot analysis and IHC assay confirmed that overexpression of ZEB1 significantly abrogated the gemcitabine-induced elevation of p-γH_2_AX and cleaced-PARP-1 in ROCK2-deletion tumor (Figure 7F,G). In accordance with the results in vitro, ROCK2, p-p38, p-sp1, and ZEB1 expression were also downregulated in ROCK2-deletion or fasudil treatment groups compared with control group (Figure 7F). Collectively, ZEB1 is critical for the resistance of the SW1990/GEM cell-driven xenograft tumor to gemcitabine conferred by ROCK2.

## 4. Discussion 

Currently, PDAC is the fourth leading cause of cancer-related mortality in the world due to the lack of accurate biomarkers for early diagnosis and effective treatment [1,33,34]. Gemcitabine, as the first-line agent for PDAC, has also achieved favorable therapeutic effects in other cancers. However, the severe systemic toxicity caused by a high dose of gemcitabine limits the application of gemcitabine and reduces the chemotherapeutic effect of gemcitabine. What is more, gemcitabine resistance induced by complicated tumor microenvironment [35] and high metastasis characteristic is the main reason for clinical failure in PDAC. 

ROCK2 has been reported to mediate the chemoresistance in some cancers, however, the potential mechanism of ROCK2 in regulating chemotherapy resistance in pancreatic cancer is still unclear. In our study, we found that GR cells exhibited stronger ROCK2 activity compared with parental cells. The enhanced cytotoxicity of gemcitabine in response to ROCK2-depletion highlighted the decisive role of ROCK2 on the resistance of gemcitabine.

Extensive studies have shown that chemotherapy-resistant cancer cells may undergo EMT. Mesenchymal proteins are usually overexpressed and mediated chemoresistance in a variety of cancers [36,37]. The overexpression of mesenchymal proteins in GR cells prompted us to focus on exploring the mesenchymal proteins-induced resistance of GR cells to gemcitabine and found that ZEB1 was the dominant factor inducing gemcitabine resistance in GR cells. Interestingly, GR shROCK2 cells colonies expressed a low level of ROCK2 and high level of Ecadherin after gemcitabine treatment. We hypothesized that the doses of gemcitabine are too low to kill all GR cells, or that a small number of cells probably survive by regulating other resistance pathways (Supplementary Figure S15). However, not all the EMT-related chemoresistance is mediated by ZEB1, as reported that paclitaxel resistance in many cancer cells are conferred by overexpression of Twist [38,39]. It seems that chemoresistance in different cancer cells or induced by different chemotherapeutic drugs may depend on different mesenchymal proteins.

Rho/ROCK signaling is considered to play part in tumor cell invasion and migration. However, it is not clear which mesenchymal markers are regulated by ROCK2 signaling in GR cells. In our study, inhibition of ROCK2 activity decreased the expression of mesenchymal proteins such as Snail, Vimentin, and ZEB1 and increased the expression of E-cadherin. However, ROCK2 inhibition had no significant effect on the expression of Slug, Twist, and Fibronectin, indicating that additional upstream effectors are required to induce EMT in GR cells.

In our study, we first discovered ROCK2 induced ZEB1-conferred gemcitabine resistance in pancreatic cancer cells. However, their regulatory mechanism remained to be fully understood. We observed that both Snail and ZEB1 were downregulated upon ROCK2 inhibition in GR cells. As reported that expression of some mesenchymal proteins, including ZEB1 can be regulated by snail in some cases [40]. However, ROCK2 regulated the expression of ZEB1 in a Snail-independent manner in both parental and GR cells. Therefore, we focused on exploring other potential signaling pathways that involved in regulation of ZEB1 by ROCK2. Current researches show that both p38 and sp1 are closely associated with EMT process [41,42]. However, it is unclear whether p38/sp1 signaling pathway is required for ROCK2 mediated EMT in GR cells. In our study, ROCK2 was found to be involved in p38-induced activation of sp1 in GR cells. The results of CHIP assay further confirmed that ROCK2-induced expression of ZEB1 was p38/sp1 dependent. More specifically, we found three potential sp1 binding sites on the ZEB1 promoter. Our results further show that ZEB1-activated ATM/p-CHK1-mediated DNA repair [43,44] contributed to gemcitabine resistance of pancreatic cancer cells.

**Figure 8:**
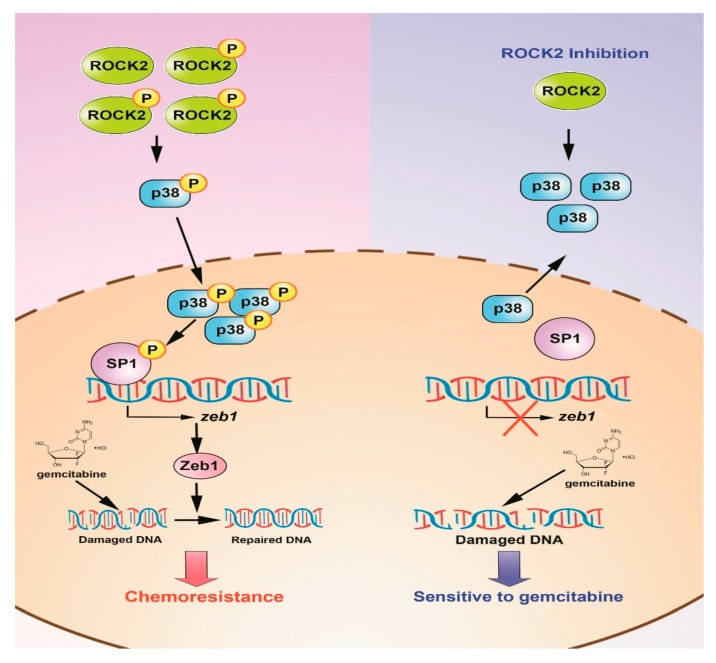
The signaling pathway involved in GR cells with ROCK2 inhibition. In GR cells, phosphorylated ROCK2 activated p38 and promoted p-p38 translocated into the nucleus. P-p38 enhanced ZEB1 transcription through phosphorylating sp1 and promoting sp1 binding to the ZEB1 promoter region. ZEB1 decreased sensitivity of pancreatic cancer cells by repairing gemcitabine-mediated DNA damage. However, when ROCK2 signal was inhibited, p38/sp1/ZEB1 signaling was blocked, and ZEB1-induced gemcitabine resistance of GR cells was reversed.

Targeting EMT to overcome chemotherapeutic drug resistance seems to be a feasible strategy [37,45,46], although it has not been successfully transformed into clinical practice. In our study, ROCK2 signaling pathway desensitizes GR cells to gemcitabine through ZEB1-mediated DNA damage repair. ROCK2-mediated upregulation of ZEB1 is closely associated with the resistance of gemcitabine-induced DNA damage. Besides, treatment with gemcitabine plus fasudil produces a satisfactory reversal effect on gemcitabine resistance, which agrees with Vennin C et al. where they found that transient ROCK inhibition by fasudil improves the efficacy of Gem/Abraxane in KPC mouse-driven and patients-driven primary PC cells [12]. They also pointed out that fasudil-mediated destruction of ECM in tumor microenvironment improves the efficacy of Gem/Abraxane. In our study, ablation of ROCK2-induced the suppression of ZEB1-dependent DNA damage homologous recombination repair is the main cause for the sensitization of GR cells to gemcitabine in vitro and in vivo (Figure 8). However, the residual gemcitabine resistance upon ROCK2 inhibition indicates the possibility of other potential factors inducing gemcitabine resistance. Furthermore, p-ROCK2 upregulates ZEB1 expression through activating the p38/sp1 signaling pathway. The different characteristics between acquired gemcitabine-resistant cells and primary tumor cells may elucidate the diversification of drug resistance mechanisms. Taken together, we proposed that the ROCK signal in pancreatic tumor tissues and surrounding matrix might coordinate with each other to maintain the resistance of pancreatic cancer cells to chemotherapeutic drugs. 

In summary, inhibition of ROCK2 increased the sensitivity of GR cells to gemcitabine. Furthermore, ZEB1 played an essential role in ROCK2-mediated gemcitabine resistance, and ROCK2 upregulated ZEB1 transcription through activation of p38/sp1 signaling pathway. Importantly, treatment with fasudil enhanced the therapeutic effect of pancreatic cancer cells in response to gemcitabine treatment, which might provide a possible clinical solution for gemcitabine-resistant PDAC.

## 5. Conclusions

Our results suggest that the high expression of phosphorylated ROCK2 causes ZEB1-mediated gemcitabine resistance in pancreatic cancer cells. Importantly, inhibition of ROCK2 signal sensitizes GR cells to gemcitabine-mediated DNA damage. Mechanically, a novel sp1/p38/ZEB1 regulatory network was found to be involved in ROCK2-mediated gemcitabine resistance in pancreatic cancer cells. The inhibition of the ROCK2 signal combined with gemcitabine treatment might appear to be a promising approach against gemcitabine resistance in pancreatic cancer. 

## Figures and Tables

**Table 1:** Primer for PCR assay.

Gene	Forward (5′-3′)	Reverse (5′-3′)
ROCK1	GGGCGAAATGGTGTAGAAGA	AATCGGGTACAACTGGTGCT
ROCK2	TGGATGAAACAGGCATGGTA	CATTCTCGCCCATAGAAACC
GAPDH	TGGTATCGTGGAAGGACTCA	CAGTAGAGGCAGGGATGATG
E-cadherin	ACCATTAACAGGAACACAGG	CAGTCACTTTCAGTGTGGTG
Vimentin	CGCCAACTACATCGACAAGGTGC	CTGGTCCACCTGCCGGCGCAG
ZEB1	GGCATACACCTACTCAACTACGG	TGGGCGGTGTAGAATCAGAGTC
ZEB2	AATGCACAGAGTGTGGCAAGGC	CTGCTGATGTGCGAACTGTAGG
Slug	TTCGGACCCACACATTACCT	GCAGTGAGGGCAAGAAAAAG
snail	TGCGCGAATCGGCGACCC	CCTAGAGAACCGCTTCCCGCAG
Twist	GGAGTCCGCAGTCTTACGAG	TCTGGAGGACCTGGTAGAGG
Fibronectin	CAGGATCACTTACGGAGAAACAG	GCCAGTGACAGCATACACAGTG

**Table 2:** Primers to detect purified DNA in ChIP assay.

Gene	Forward (5′-3′)	Reverse (5′-3′)
F1	CATGGCCTGTGGATACCTTAG	CTGGATTGAAAGAGAGGCTAGAA
F2	CTTATTCGAAGGAGGTGGGAAG	GCAGGACCTTAAGGCAAGAA
F3	AATCCTGCCATAGAAGTGACAAA	GGGACCAACTTTATGGAATAAATAAGC
F4	TGAGGATGAATGCAGATATATAGAC	ATGTCTTCAAACCTTTCAACTG
F5	CTGGTCAGAAATCAGGGTAGCTG	GGCAGTCCTCGCTTTCCTTG
F6	ACTTGTCCACAGTTTGGCCC	TCCAGCTCTATCACACATTTTACCT
F7	GGTGAACAGAGTTCATTGTTTAGG	TGGAGTACGTAGCCAATAGTAGA
F8	GAGATAAGAAGCAACCGTCACA	ACTGGTAGCCCAAATCTTCTAAC

**Table 3:** Effect of fasudil on the sensitivity of GR cells to gemcitabine.

Group	Concentration (μM)	SW1990/GEM IC50 of Gemcitabine (μM)	RF ^a^	Panc-1/GEM IC50 of Gemcitabine (μM)	RF ^a^
Fasudil	0	621.35 ± 20.75	1	293.50 ± 37.25	1
	0.5	517.84 ± 34.75	1.20	199.09 ± 36.99	1.47
	2.0	147.59 ± 27.74	4.21	97.44 ± 19.59	3.01
	8.0	28.69 ± 4.96	21.66	24.23 ± 2.75	12.11

^a^ Reversal fold.

**Table 4:** Synergism of fasudil and gemcitabine in GR cells.

Fasudil (μM)	Gemcitabine(μM)	SW1990/GEM		Panc-1/GEM	
		Effect	CI ^a^	Effect	CI ^a^
0.5	15	0.1252	0.25983	0.1279	0.53052
	30	0.1763	0.31237	0.2039	0.53637
	60	0.2588	0.34413	0.3065	0.55136
	120	0.3172	0.48717	0.4082	0.64082
	240	0.3915	0.6595	0.5242	0.7241
2	15	0.1355	0.26857	0.1451	0.44657
	30	0.2249	0.23109	0.2315	0.44054
	60	0.3481	0.21289	0.411	0.31596
	120	0.4275	0.2801	0.5872	0.26514
	240	0.5832	0.26274	0.6665	0.35032
8	15	0.3229	0.09433	0.3876	0.08922
	30	0.5721	0.04195	0.5809	0.06844
	60	0.6364	0.05499	0.6856	0.07875
	120	0.7912	0.04142	0.7626	0.09821
	240	0.9802	0.00377	0.9128	0.04652

^a^ CI values were calculated by CalcuSyn software, and drug interactions were indicated. as synergism (CI < 0.9), additivity (0.9 < CI< 1.1) or antagonism (CI > 1.1).

**Table 5:** Sensitization of GR cells to different anticancer drugs induced by fasudil.

Group		SW1990/GEM		Panc-1/GEM	
		IC_50_	RF ^a^	IC_50_	RF ^a^
5-FU	Control	191.02 ± 16.93	1	181.76 ± 25.45	1
	Fasudil 8 μM	64.56 ± 6.00	2.96	74.76 ± 9.48	2.43
Paclitaxel	Control	348.44 ± 39.75	1	196.27 ± 10.29	1
	Fasudil 8 μM	119.30 ± 12.14	2.92	125.04 ± 20.43	1.57
Cisplatin	Control	195.36 ± 11.64	1	171.54 ± 16.80	1
	Fasudil 8 μM	87.52 ± 2.36	2.23	69.15 ± 5.64	2.48
Gemcitabine	Control	621.35 ± 20.75	1	294.50 ± 37.25	1
	Fasudil 8 μM	2869 ± 4.96	21.66	24.23 ± 2.75	12.15

^a^ Reversal fold.

## Supplementary Materials

The following are available online at https://www.mdpi.com/2072-6694/11/12/1881/s1, Figure S1: The effect of gemcitabine stress and gemcitabine selection mechanism on ROCK2 expression in pancreatic cancer cells, Figure S2: Synergism of fasudil and gemcitabine in parental cells, Figure S3: CI values of fasudil combined with a 5-FU, b PTX and c Cis in GR cells, respectively, Figure S4: Effect of fasudil and gemcitabine on phosphorylation of ROCK2 in GR cells, Figure S5: Knockdown efficiency of shRNA in GR cells. western blot was used to detect the expression of ROCK2 upon ShROCK2#1 and ShROCK2#2 transfected into GR cells, respectively, Figure S6: Knockdown of ROCK2 enhances GEM-induced inhibition of cell proliferation, Figure S7: The effect of EMT on ROCK2 expression in pancreatic cancer cells, Figure S8: Fasudil reverses abnormal expression of EMT markers in parental cells, Figure S9: Detection of ZEB1 overexpression efficiency, Figure S10: ZEB1 is a key factor in ROCK2-mediated GR cells resistance to apoptosis induced by chemotherapy drugs, Figure S11: ZEB1 is a key factor in ROCK2-mediated GR cells resistance to chemotherapy drugs, Figure S12: Regulation of ZEB1 by ROCK2 is independent of snail. Protein expression levels were detected by western blot, Figure S13: The expression of p-p38 and p-sp1 in GR cells in GR cells and parental cells, Figure S14: ROCK2 mediates Sp1-induced upregulation of ZEB1 promoter luciferase activity, Figure S15: The protein expression of ROCK2 and Ecadherin in cell clones upon GEM treatment. Raw data of Western blot.
