# Supplementary material for: ROCK2 Confers Acquired Gemcitabine Resistance in Pancreatic Cancer Cells by Upregulating Transcription Factor ZEB1
Source: Cancers (Basel). 2019 Nov 27;11(12):1881. doi: 10.3390/cancers11121881 (PMC6966455; doi:10.3390/cancers11121881)
Supplement: Supplementary file 1 [file cancers-11-01881-s001.pdf]

cancers

Supplementary Materials: ROCK2 Confers Acquired Gemcitabine Resistance in Pancreatic Cancer Cells by Upregulating Transcription Factor ZEB1

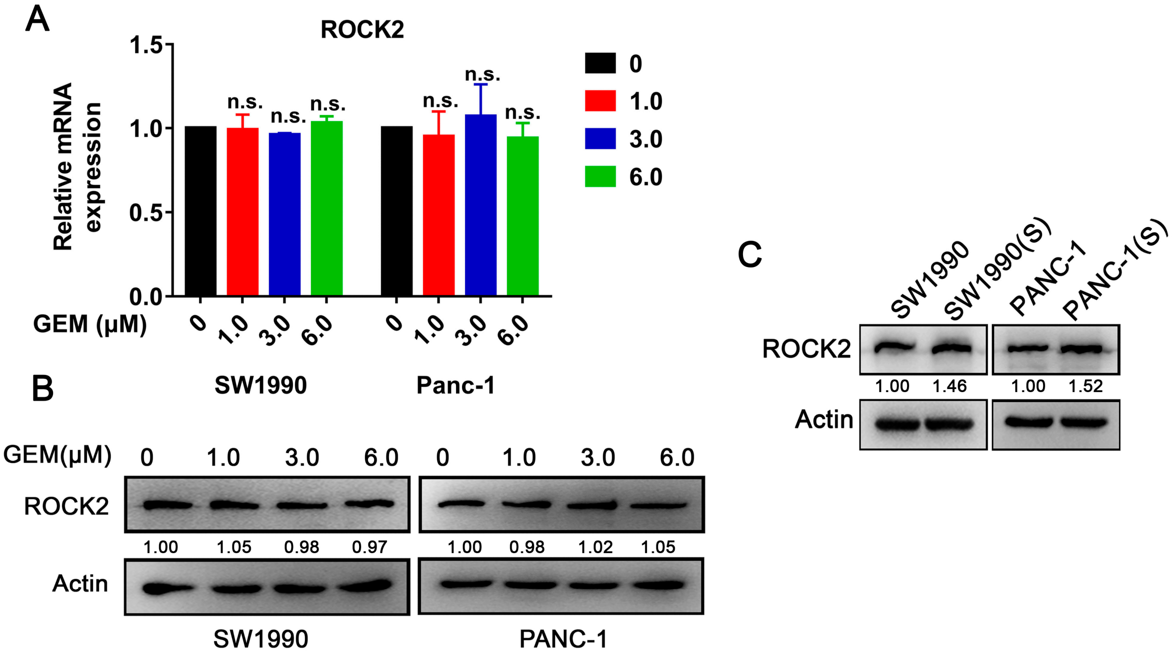

Figure S1. The effect of gemcitabine stress and gemcitabine selection mechanism on ROCK2 expression in pancreatic cancer cells. Parental cells were treated with indicated doses of gemcitabine for 24h and then the mRNA and protein expression of ROCK2 were determined by **a** real-time PCR and **b** western blot. **c** Western blot was used to detect the expression of ROCK2 in parental cells and gemcitabine selected cells which could stably grow in the medium with 5.0 μm gemcitabine (SW1990(S) and Panc-1(S)). Data represents three independent experiments (* $p < 0.05$, ** $p < 0.01$, *** $p < 0.001$).

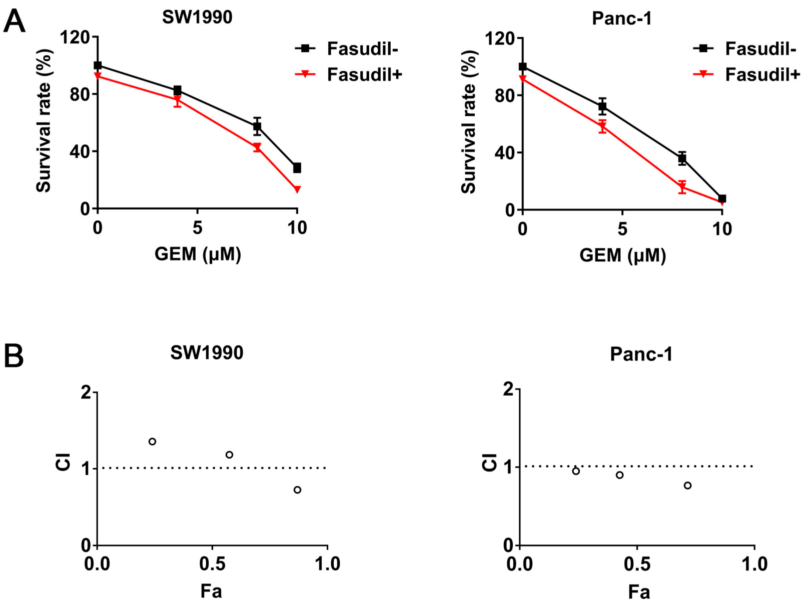

Figure S2. Synergism of fasudil and gemcitabine in parental cells. **a** MTT assay was used to verify the effect of fasudil (8μM) combined with different doses of gemcitabine on parental cells survival. **b** CI values were calculated by CompuSyn software. Data represents three independent experiments.

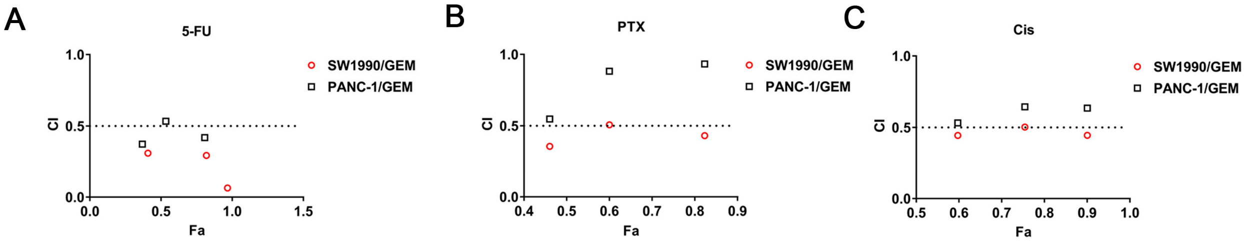

Figure S3. CI values of fasudil combined with **a** 5-FU, **b** PTX and **c** Cis in GR cells, respectively.Data represents three independent experiments.

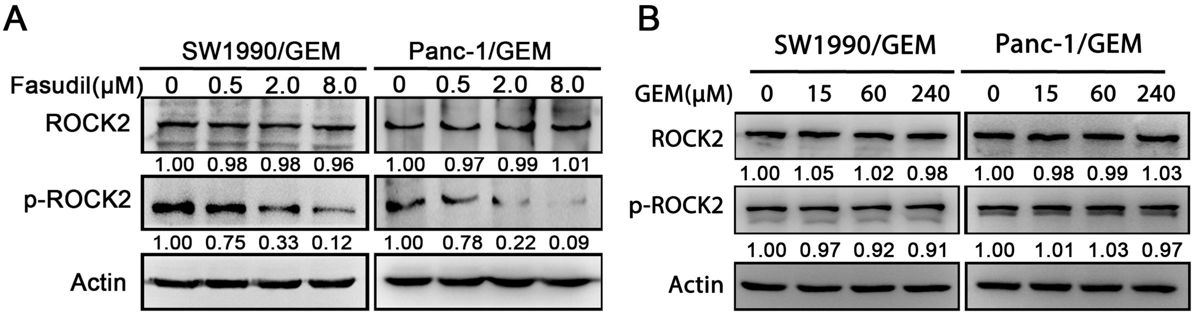

Figure S4. Effect of fasudil and gemcitabine on phosphorylation of ROCK2 in GR cells. GR cells were treated with indicated doses of fasudil or gemcitabine for 24h and then the expression of ROCK2 and p-ROCK2 were determined by western blot. Data represents three independent experiments.

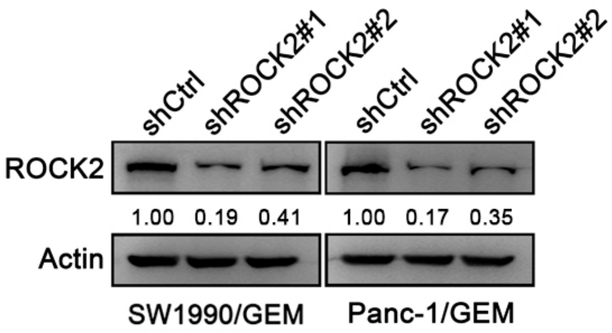

Figure S5. Knockdown efficiency of shRNA in GR cells. western blot was used to detect the expression of ROCK2 upon ShROCK2#1 and ShROCK2#2 transfected into GR cells, respectively. Data represents three independent experiments.

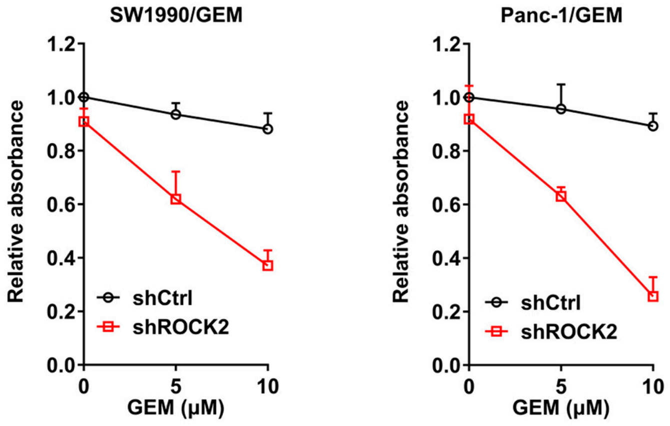

Figure S6. Knockdown of ROCK2 enhances GEM-induced inhibition of cell proliferation. ShCtrl shROCK2-GR cells were treated with different doses of GEM for 24h, BrdU incorporation assay was used to detect the cell proliferation. Data represents three independent experiments.

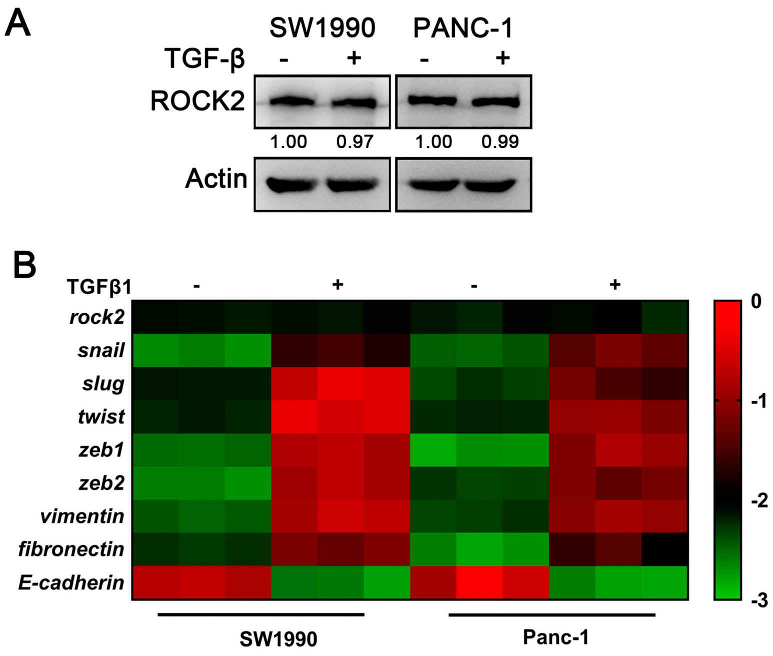

Figure S7. The effect of EMT on ROCK2 expression in pancreatic cancer cells. Parental cells were treated with TGF-β1 (2 ng/ml) for 48h. Then, protein expression of ROCK2 was detected by **a** western

blot, and the mRNA expression of ROCK2 and EMT markers was detected by **b** real-time PCR. Data represents three independent experiments.

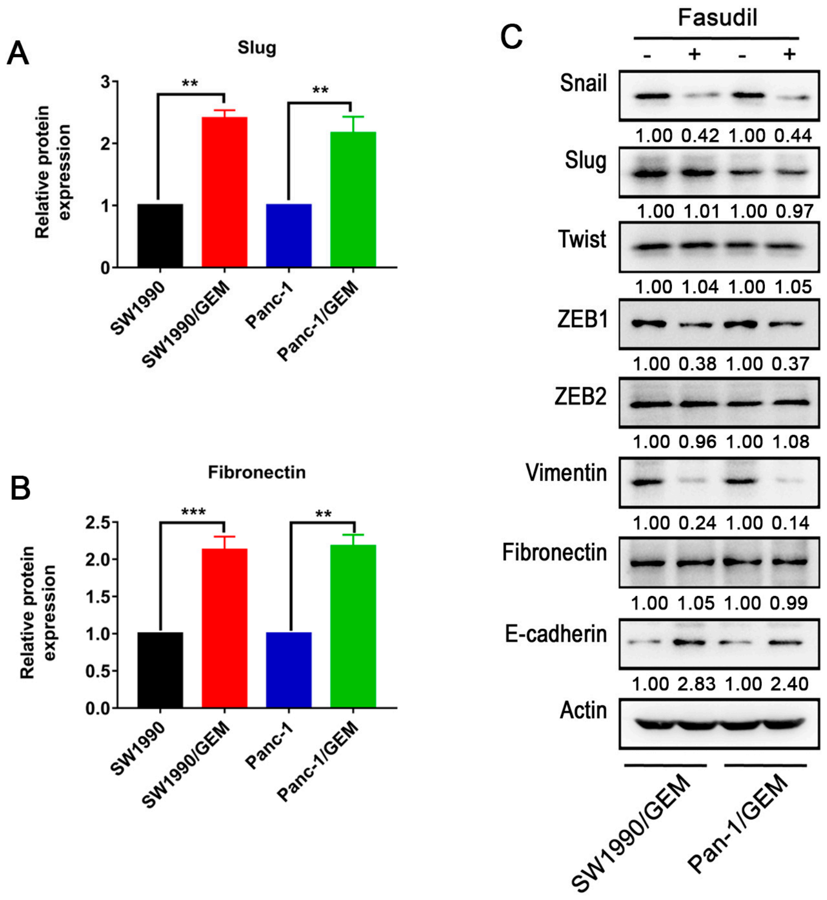

Figure S8. Fasudil reverses abnormal expression of EMT markers in parental cells. Quantification of **a** slug and **b** fibronectin expression discrepancy between parental cells and GR cells. **c** GR cells were treated with or without fasudil (8μM) for 24h and then expression levels of indicated protein were detected by western blot. Data represents three independent experiments(* $p < 0.05$, ** $p < 0.01$, *** $p < 0.001$).

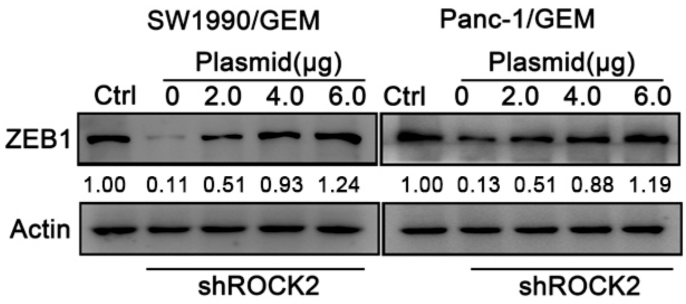

Figure S9. Detection of ZEB1 overexpression efficiency. Protein expression of ZEB1 was detected by western blot after different doses of ZEB1 plasmid were transfected into shROCK2 GR cells.

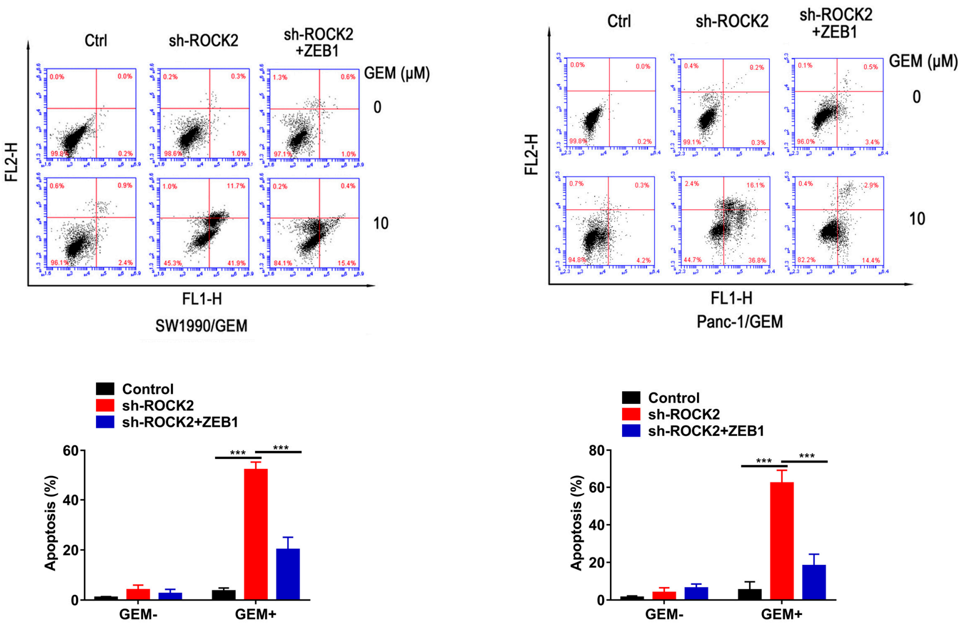

Figure S10. ZEB1 is a key factor in ROCK2-mediated GR cells resistance to apoptosis induced by chemotherapy drugs. Cells were treated with 5-FU (50μM), PTX (100nM) and Cis (100μM) for 24h. Apoptotic cells were detected by flow cytometry. Data represents three independent experiments and is presented as mean ± SD. (* $p < 0.05$, ** $p < 0.01$, *** $p < 0.001$).

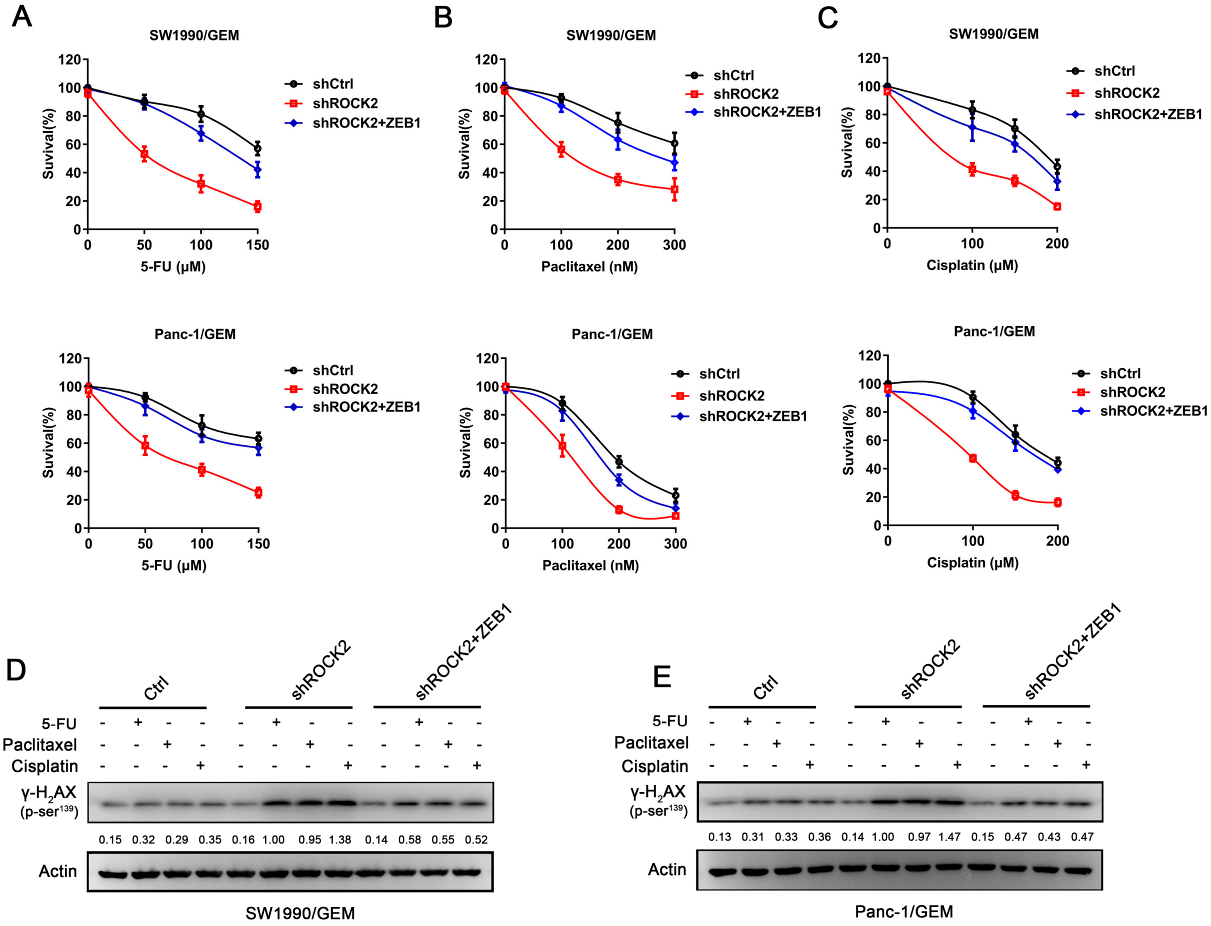

Figure S11. ZEB1 is a key factor in ROCK2-mediated GR cells resistance to chemotherapy drugs. **a**–**c** CI values of fasudil combined with 5-FU, PTX and Cis in GR cells, respectively. **a-e** Cells were treated

with 5-FU (50μM), PTX (100nM) and Cis (100μM) for 24h. **a-c** Cell viability was determined by MTT assay. **d,e** P-γH_2AX was detected by western blot. Data represents three independent experiments and is presented as mean ± SD.

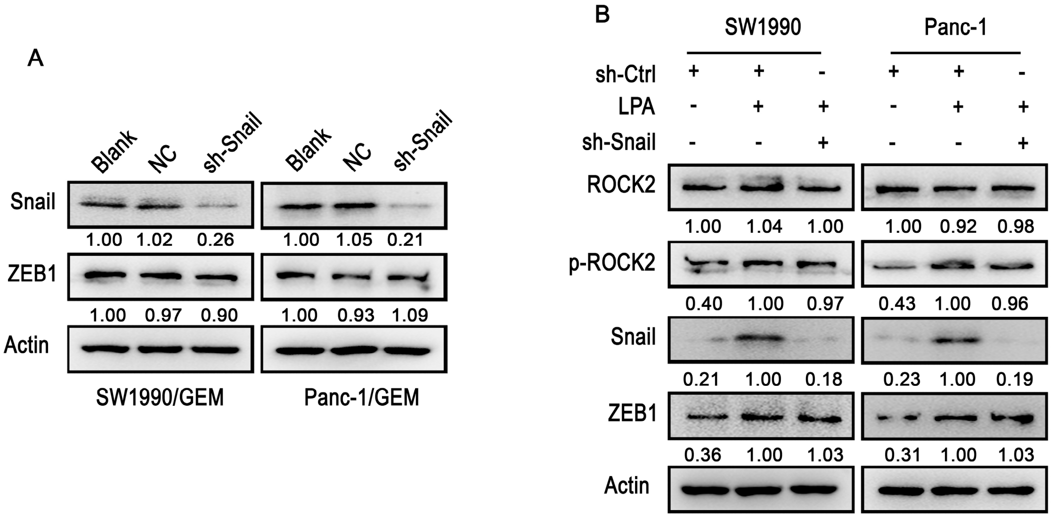

Figure S12. Regulation of ZEB1 by ROCK2 is independent of snail. Protein expression levels were detected by western blot. **a** GR cells were transfected with control shRNA or shSnail. **b** Control shRNA or shSnail were transfected into parental cells after pretreatment with LPA. Data represents three independent experiments.

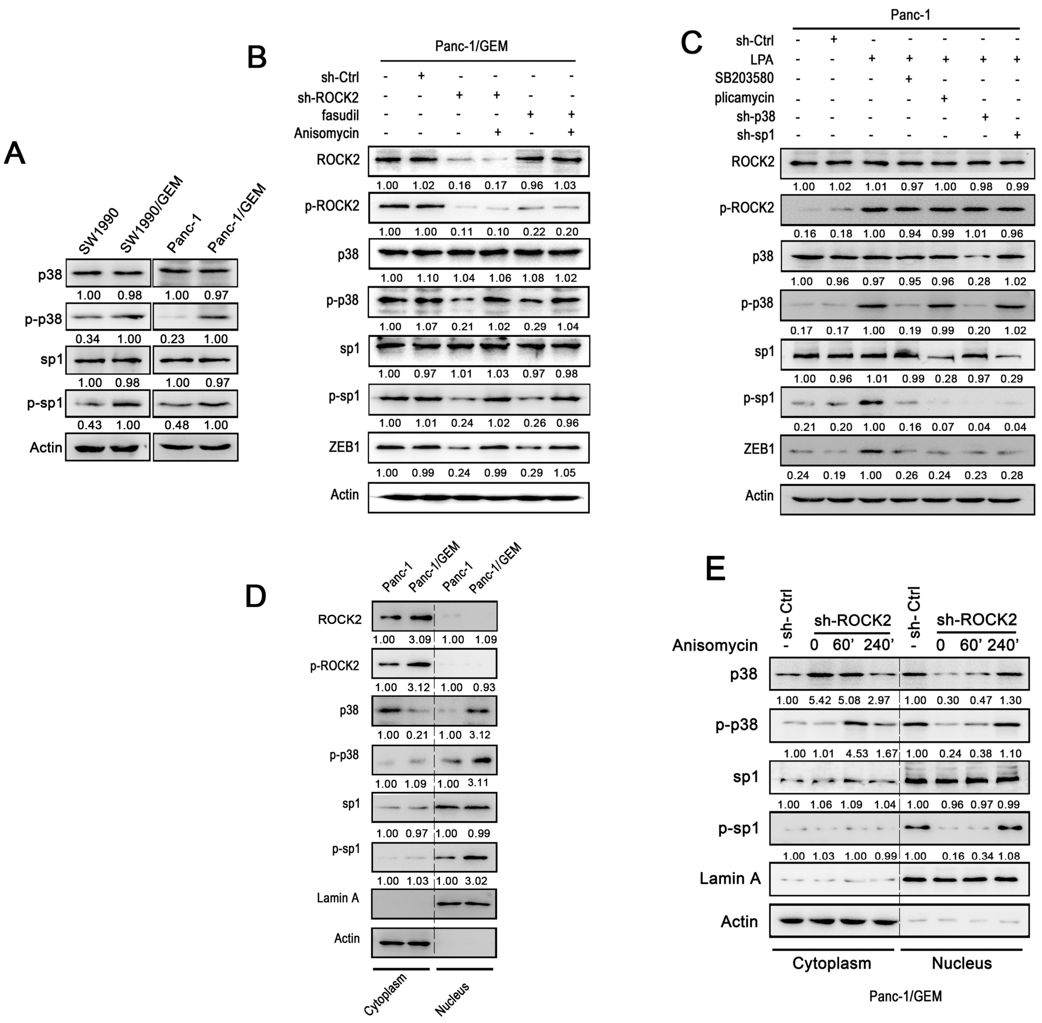

Figure S13. The expression of p-p38 and p-sp1 in GR cells in GR cells and parental cells. **a** Indicated protein expression in GR cells and parental cells were detected by western blot. **b** ShROCK2-Panc-1/GEM cell treated with or without Anisomycin. Panc-1/GEM cell treated with fasudil or Anisomycin alone or in combination. **c** Panc-1 cell was pretreated with LPA, and then treated with SB203580, plicamycin, respectively. ShCtrl, Sh-p38 or sh-sp1 were transfected into Panc-1 cell after pretreated with LPA, respectively. **d, e** Detection of the protein expression levels of p-ROCK2, p38, p-p38, sp1 and p-sp1 in nucleus and cytoplasm by western blot. **d** Differential expression of indicated proteins between GR cells and Panc-1 cell in nucleus and cytoplasm. **e** Scramble shRNA or shROCK2 were transfected into Panc-1 cell. ShROCK2-Panc-1/GEM cell was treated with Anisomycin for indicated time points. Data represents three independent experiments.

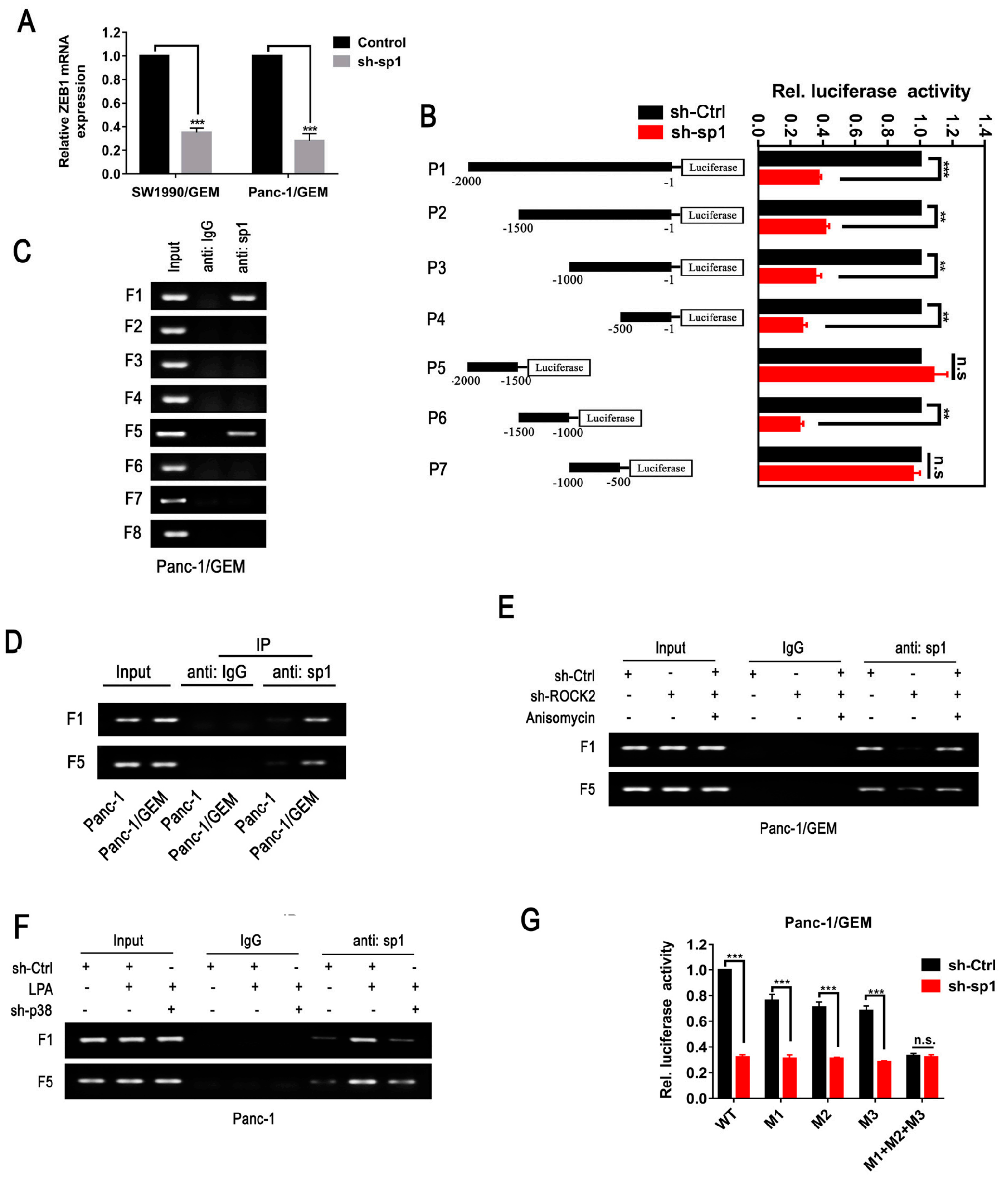

Figure S14. ROCK2 mediates Sp1-induced upregulation of ZEB1 promoter luciferase activity. **a** Relative mRNA expression of ZEB1 was detected in GR cells transfected with control shRNA or shsp1. **b** Full-size and progressively deleted ZEB1 promoter luciferase constructs were transiently transfected into Panc-1/GEM cell with or without sp1-depletion. The pRL-TK Renilla plasmid was used as an internal control, and pGL3 empty vector was used as a negative control. **c-f** Chromatin IP method was performed to detect the physical binding between sp1 and ZEB1 promoter regions. **c** Panc-1/GEM cell **d** Panc-1 cell and Panc-1/GEM cell. **e** Panc-1/GEM cell with ROCK2-ablation treated with or without Anisomycin. **f** Panc-1 cell was transfected with scramble shRNA or p38-targeting shRNA after treated with LPA. **g** Luciferase activity of ZEB1 promoter constructs with wild type or mutated binding sites for sp1 were detected in Panc-1/GEM cell and shsp1-Panc-1/GEM cell. Representative statistics derived from three independent experiments are expressed as means ± SD (* $p < 0.05$, ** $p < 0.01$, *** $p < 0.001$).

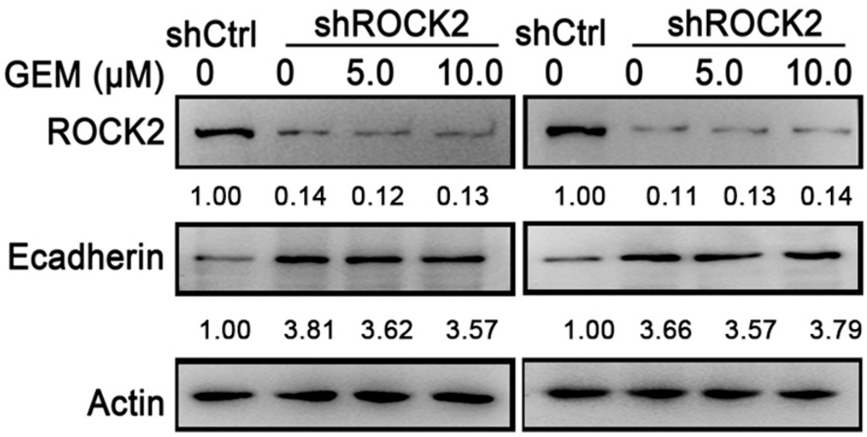

Figure S15. The protein expression of ROCK2 and Ecadherin in cell clones upon GEM treatment. Cells colonies that survived gemcitabine treatment were collected and cultured. Then, the protein expression of ROCK2 and E-cadherin in the surviving cells were detected. Data represents three independent experiments.

Raw data of Western blot

Fig. 1D

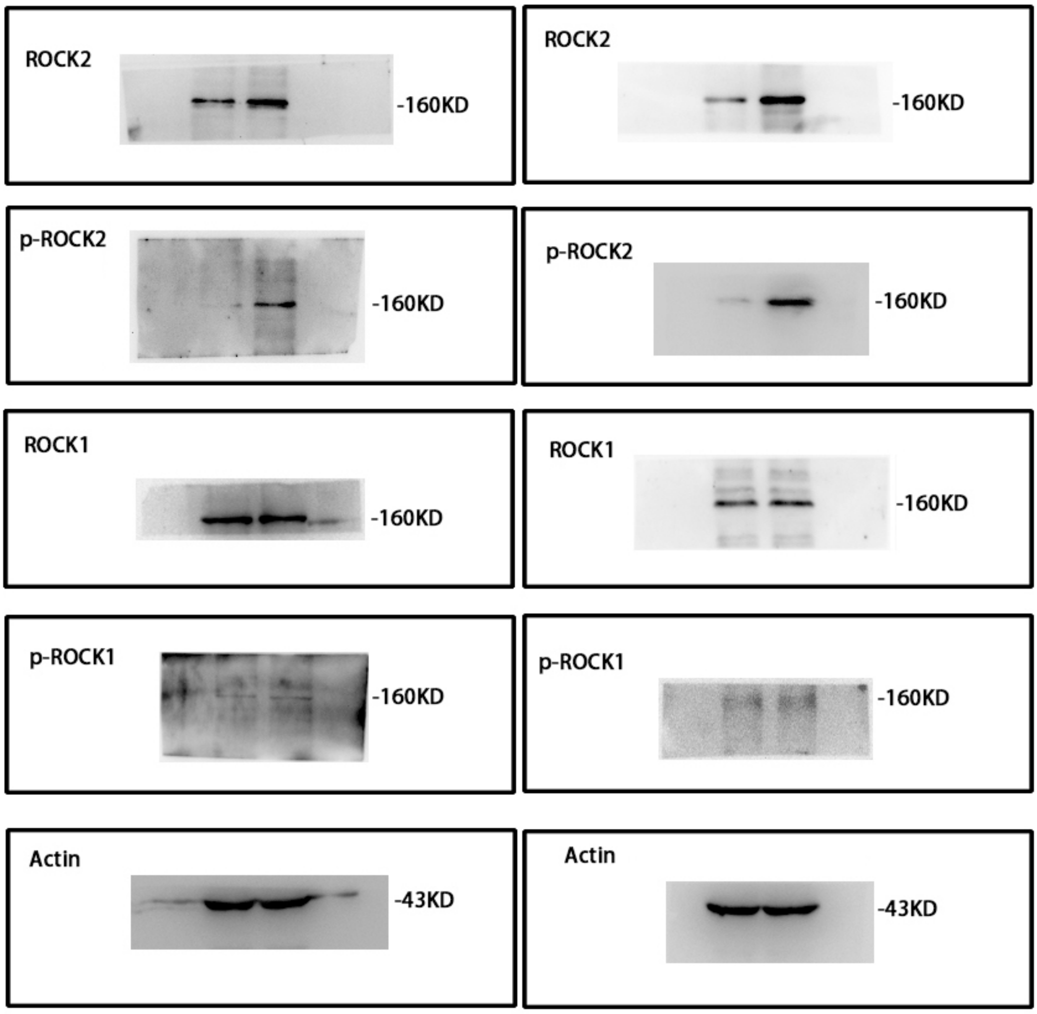

Fig. 2A

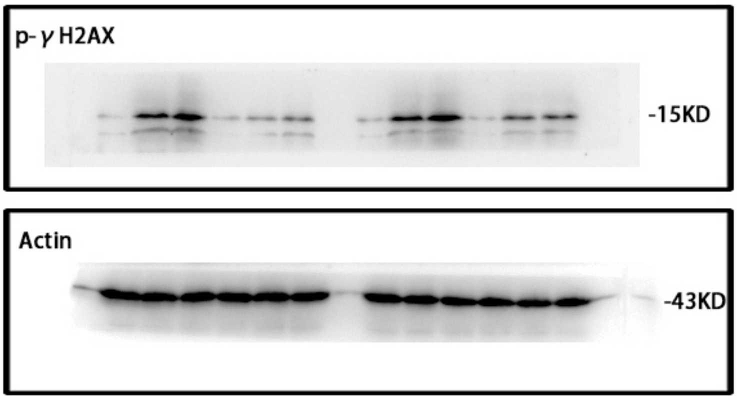
p-γ H2AX
-15KD
Actin
-43KD

Fig. 2D

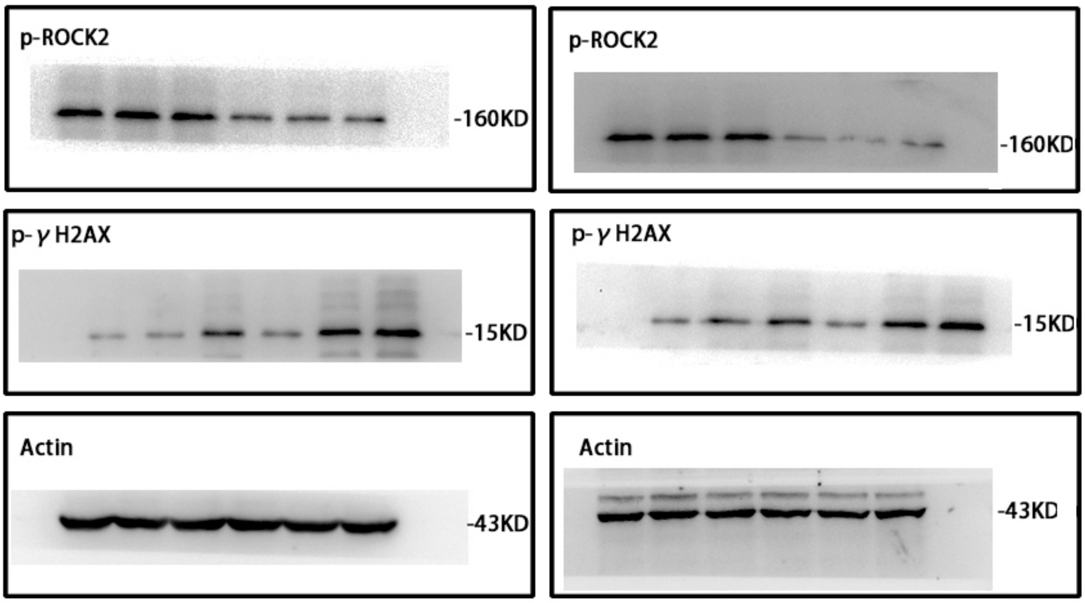
p-ROCK2
-160KD
p-ROCK2
-160KD
p-γ H2AX
-15KD
p-γ H2AX
-15KD
Actin
-43KD
Actin
-43KD

3C

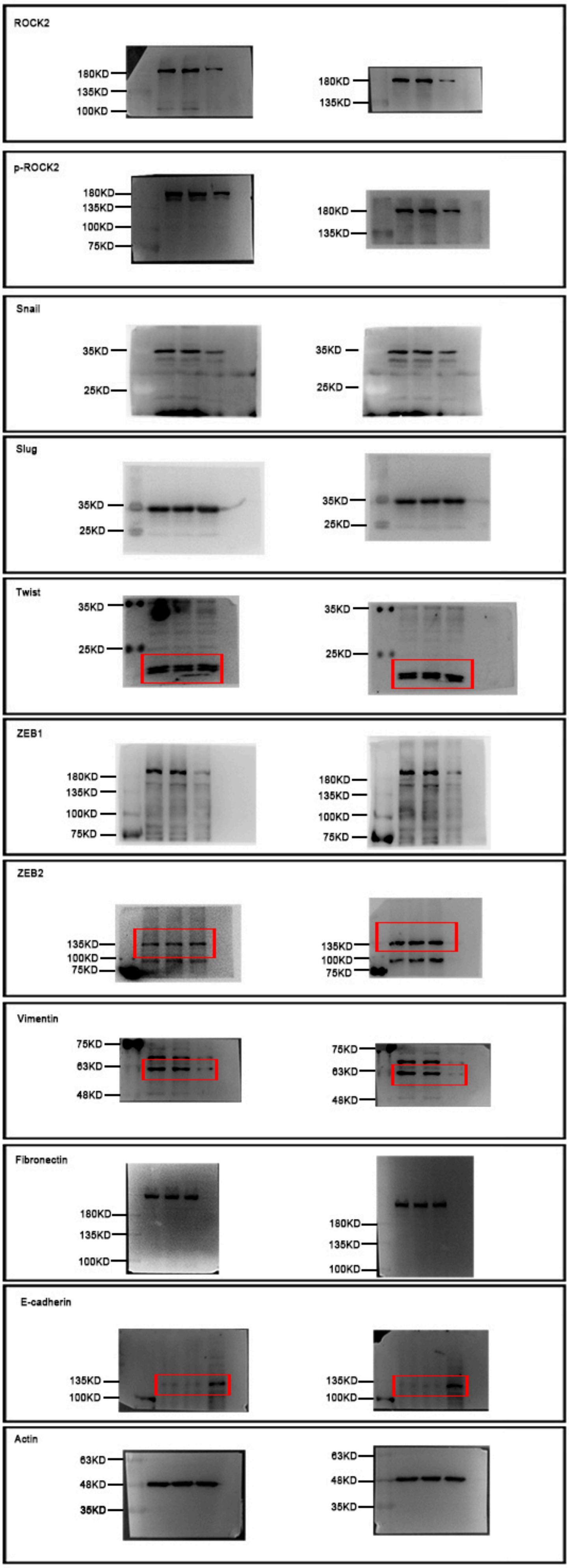
ROCK2
180KD
135KD
100KD
180KD
135KD
p-ROCK2
180KD
135KD
100KD
75KD
180KD
135KD
Snail
35KD
25KD
35KD
25KD
Slug
35KD
25KD
35KD
25KD
Twist
35KD
25KD
35KD
25KD
ZEB1
180KD
135KD
100KD
75KD
180KD
135KD
100KD
75KD
ZEB2
135KD
100KD
75KD
135KD
100KD
75KD
Vimentin
75KD
63KD
48KD
75KD
63KD
48KD
Fibronectin
180KD
135KD
100KD
180KD
135KD
100KD
E-cadherin
135KD
100KD
135KD
100KD
Actin
63KD
48KD
35KD
63KD
48KD
35KD

3B

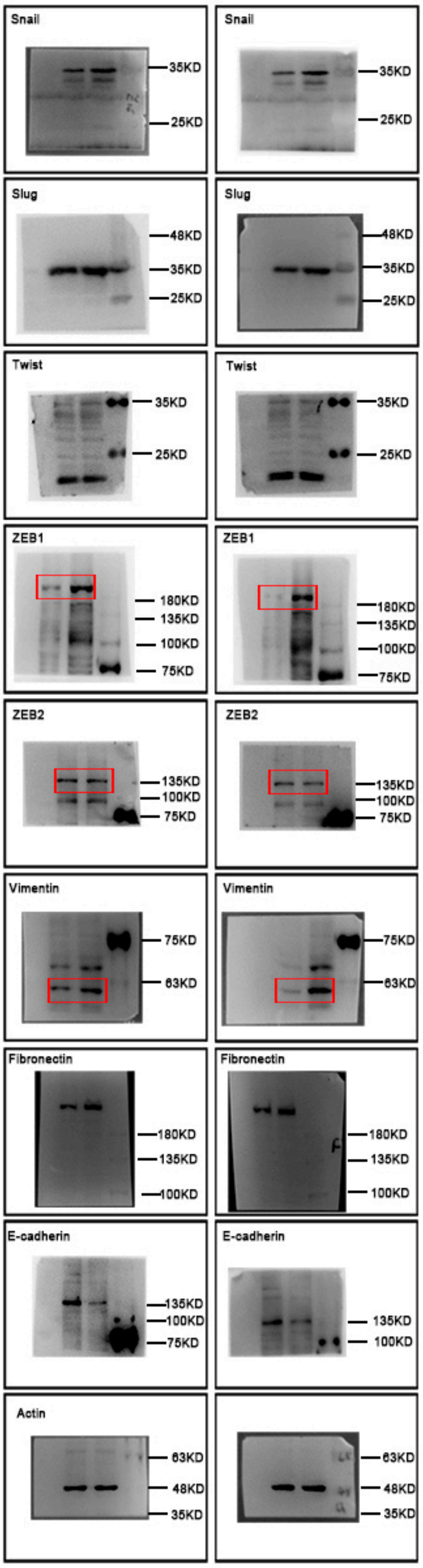
Snail
35KD
25KD
Snail
35KD
25KD
Slug
48KD
35KD
25KD
Slug
48KD
35KD
25KD
Twist
35KD
25KD
Twist
35KD
25KD
ZEB1
180KD
135KD
100KD
75KD
ZEB1
180KD
135KD
100KD
75KD
ZEB2
135KD
100KD
75KD
ZEB2
135KD
100KD
75KD
Vimentin
75KD
63KD
Vimentin
75KD
63KD
Fibronectin
180KD
135KD
100KD
Fibronectin
180KD
135KD
100KD
E-cadherin
135KD
100KD
75KD
E-cadherin
135KD
100KD
Actin
63KD
48KD
35KD
63KD
48KD
35KD

Fig. 4E

Fig. 4D

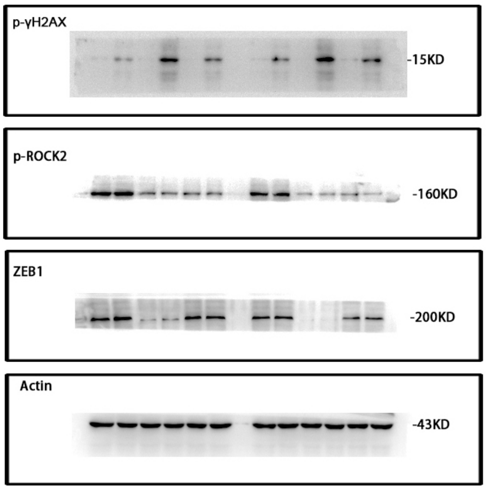

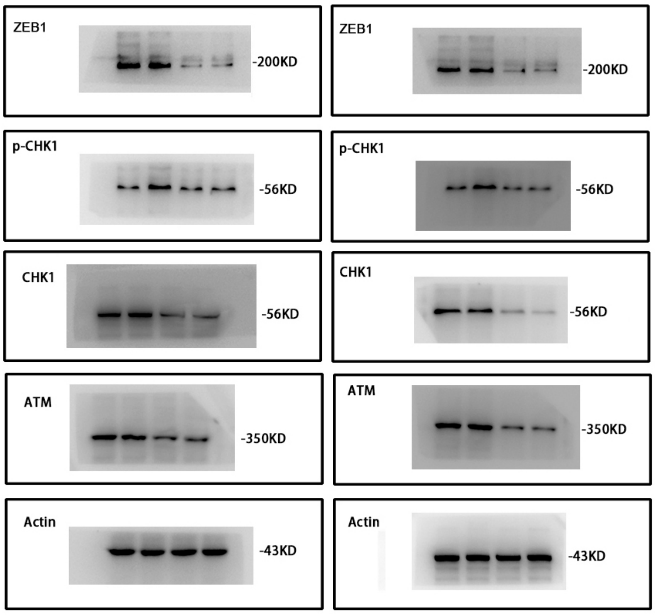

Fig. 5A

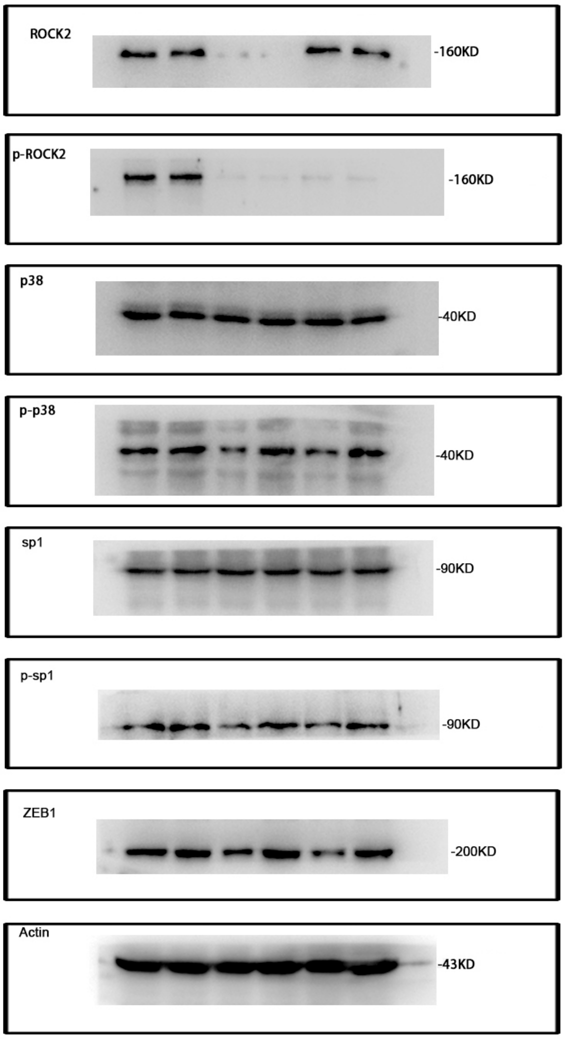

Fig. 5B

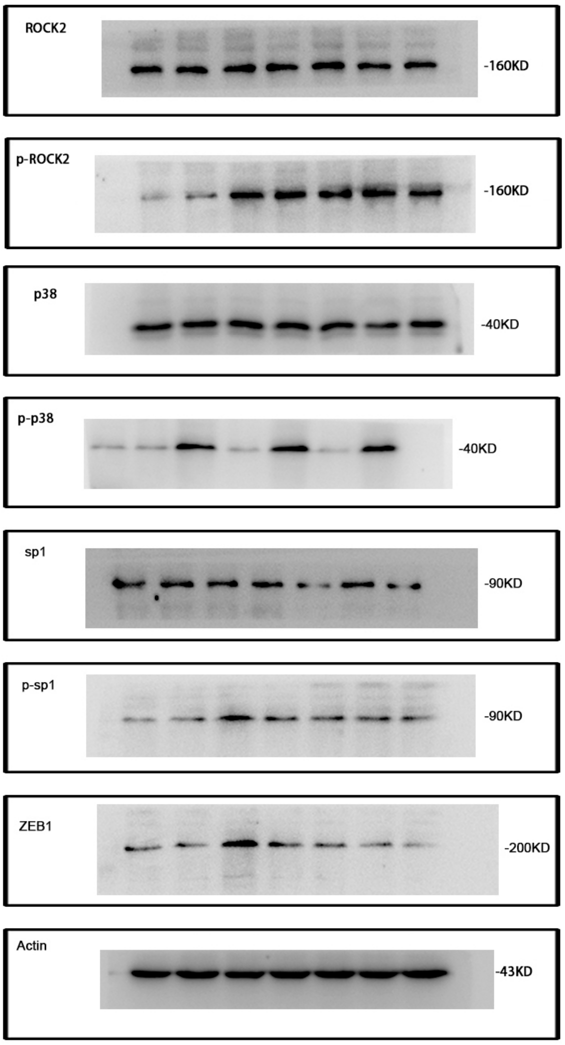

Fig. 5C

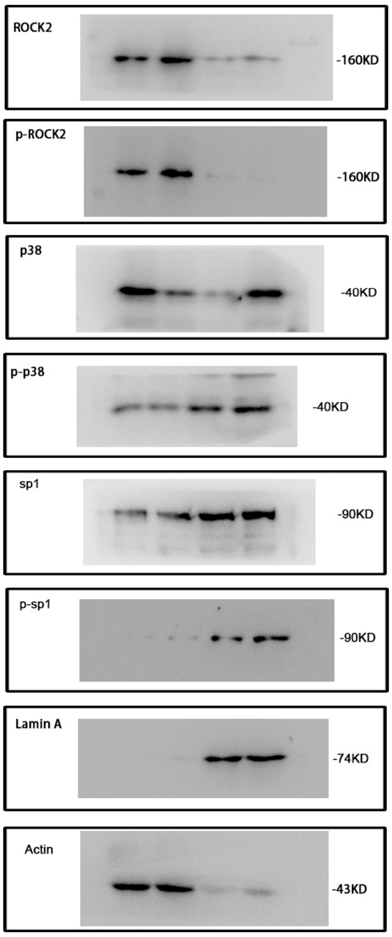

Fig. 5D

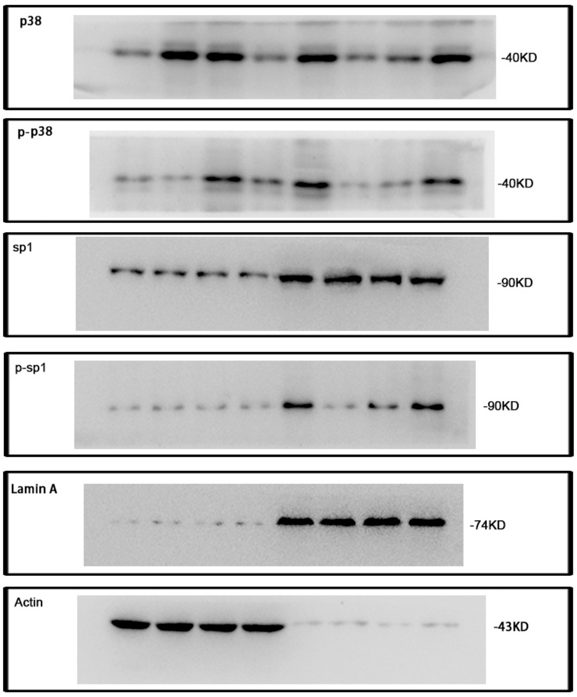

Fig. 7F

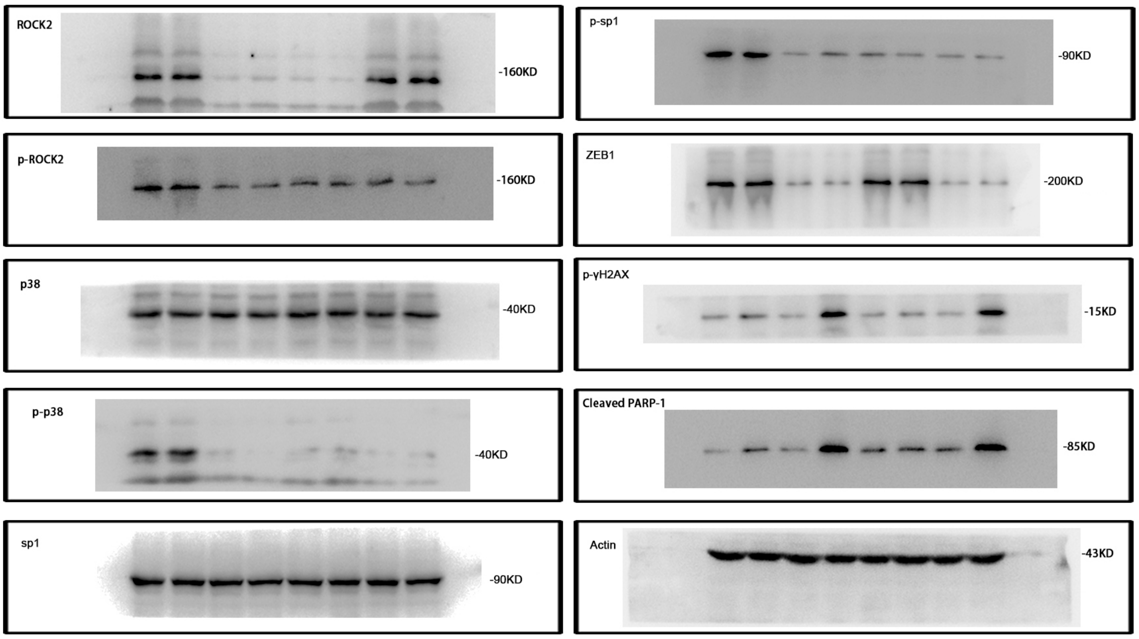

Fig. S1B

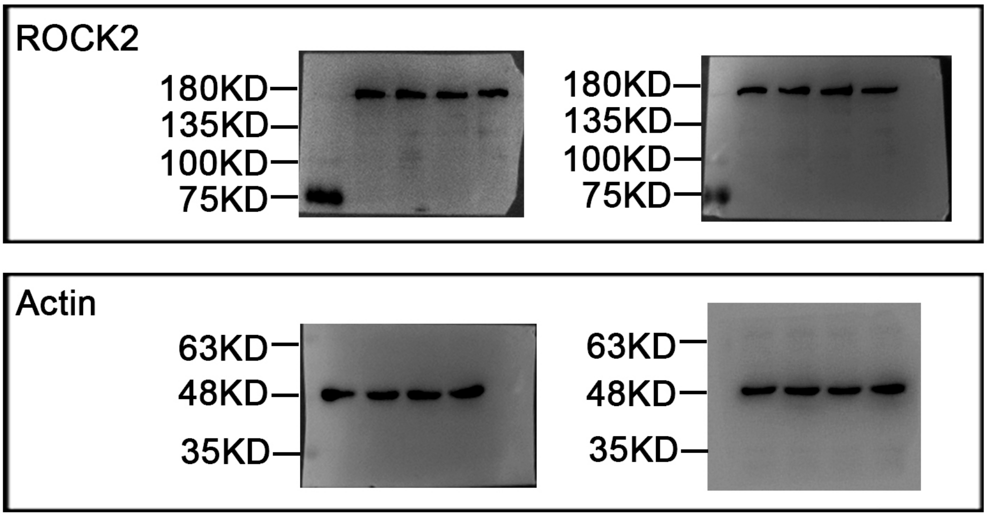

Fig. S1C

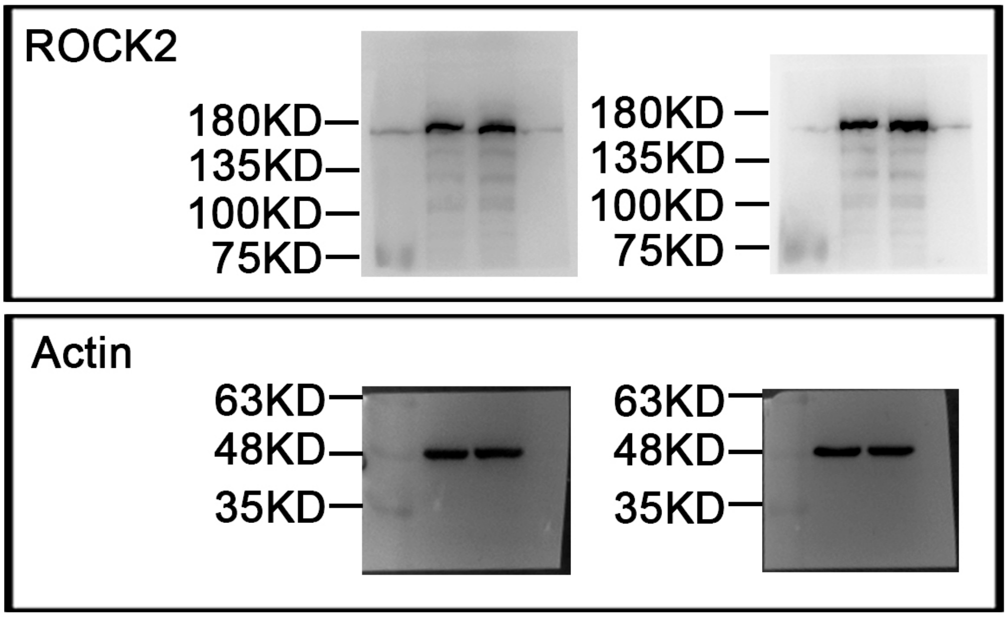

Fig. S4A

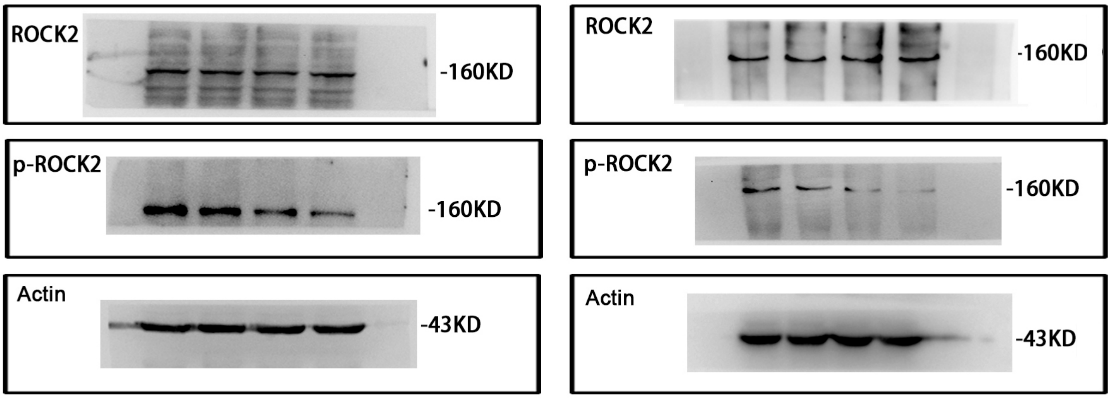

Fig. S4B

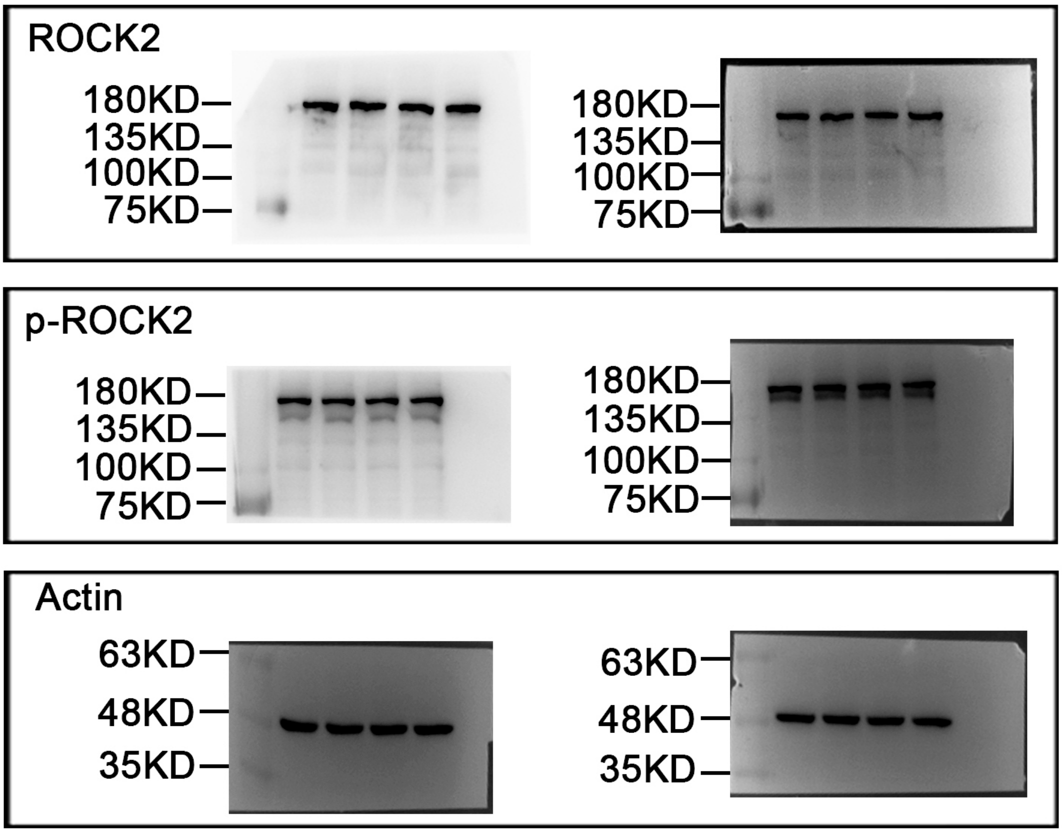

Fig. S5

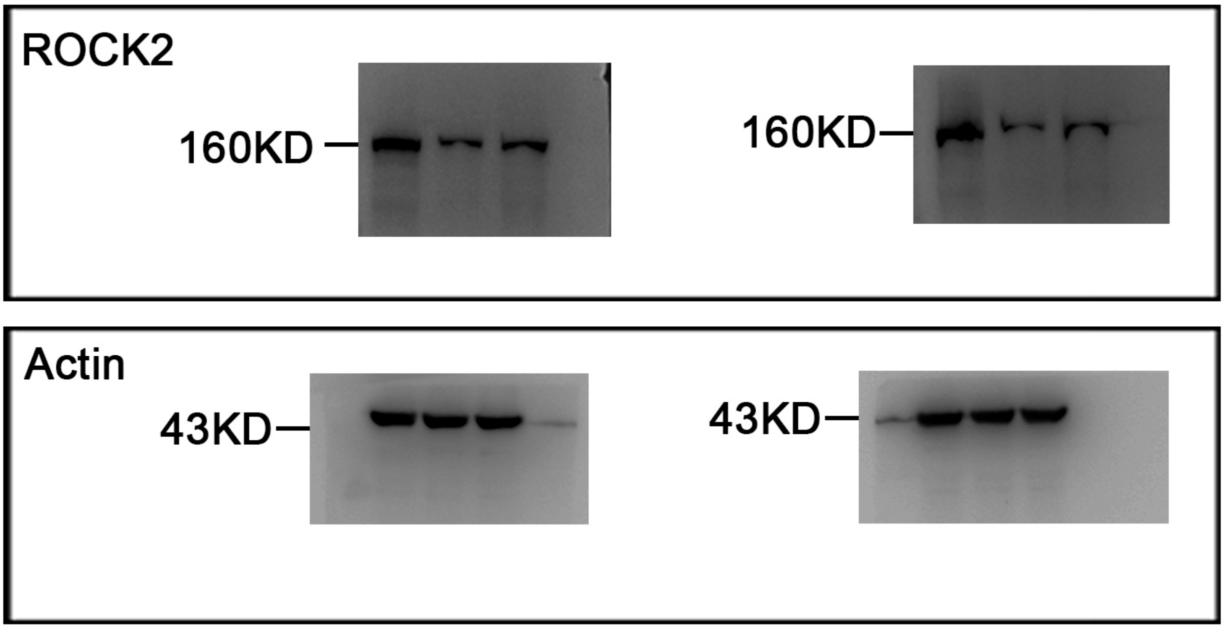

Fig. S7A

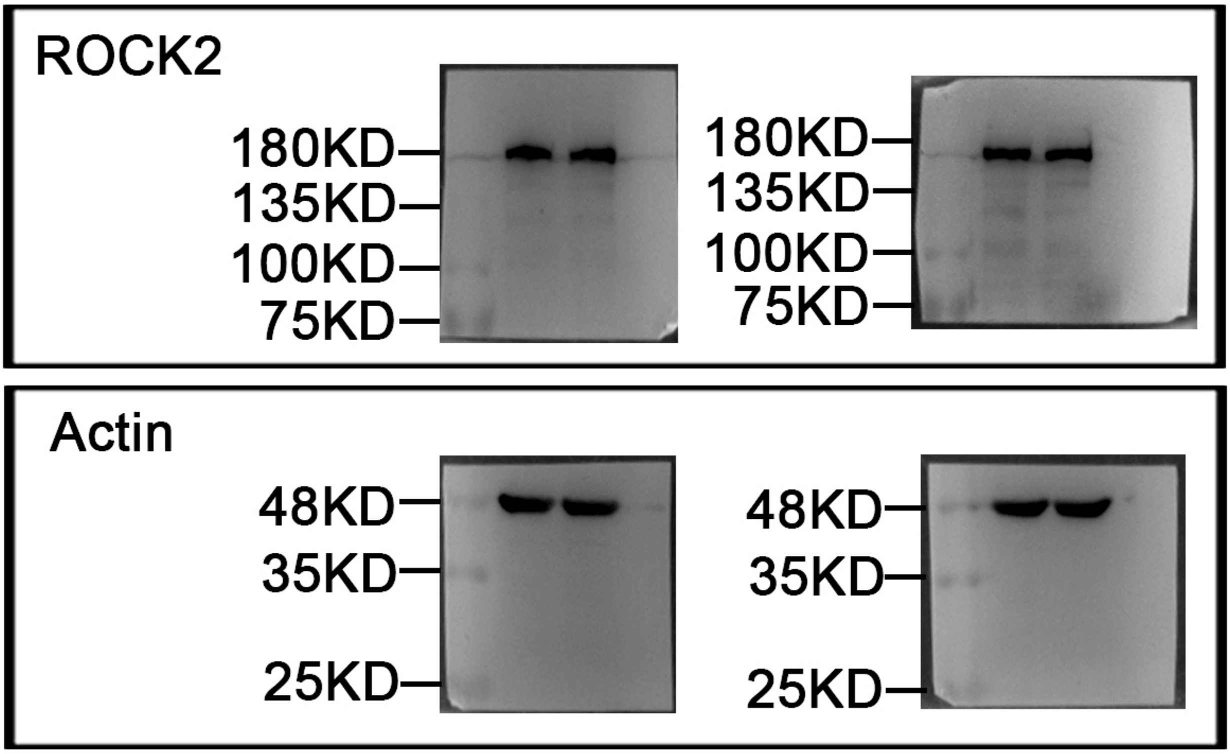

Fig. S8

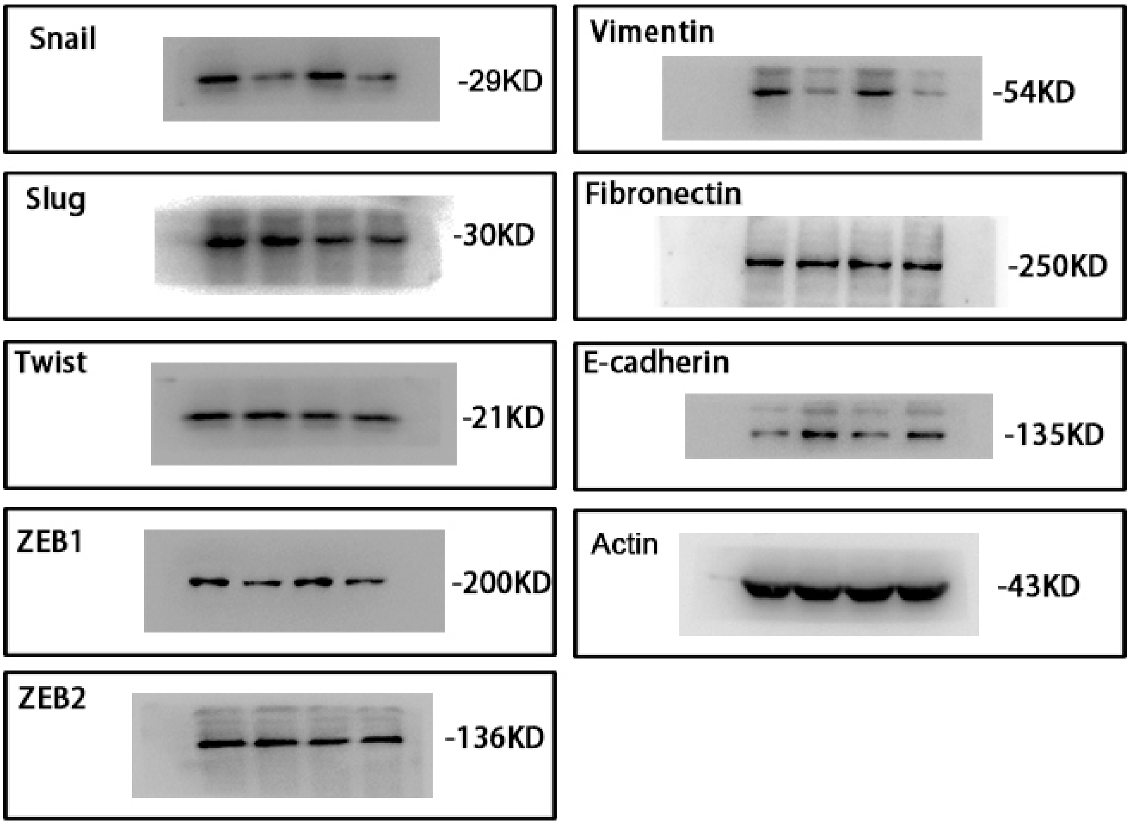
Snail
-29KD
Vimentin
-54KD
Slug
-30KD
Fibronectin
-250KD
Twist
-21KD
E-cadherin
-135KD
ZEB1
-200KD
Actin
-43KD
ZEB2
-136KD

Fig. S9

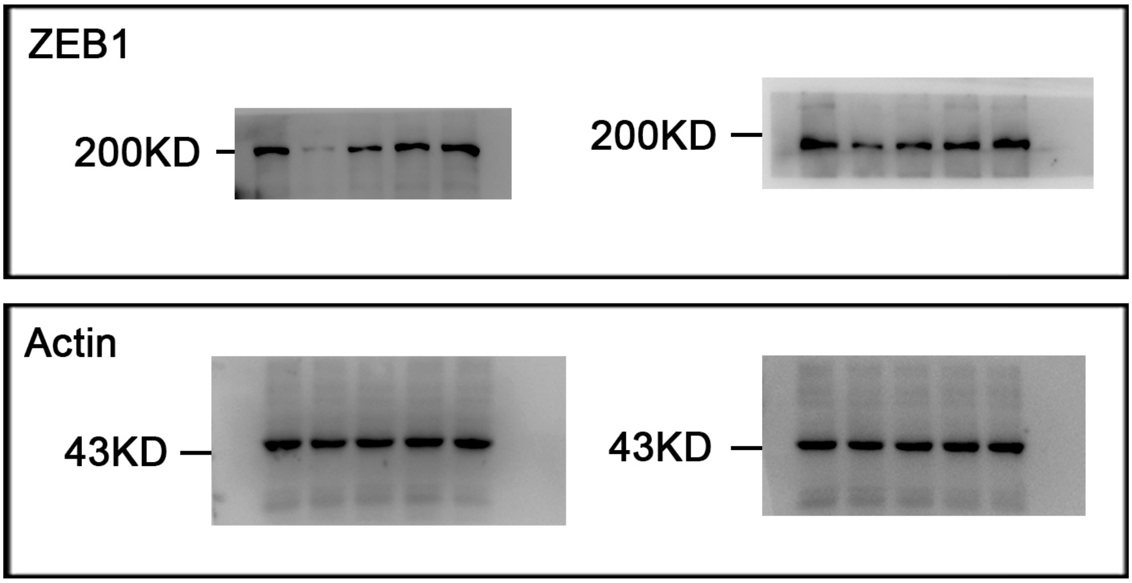
ZEB1
200KD
200KD
Actin
43KD
43KD

Fig. S11D, E

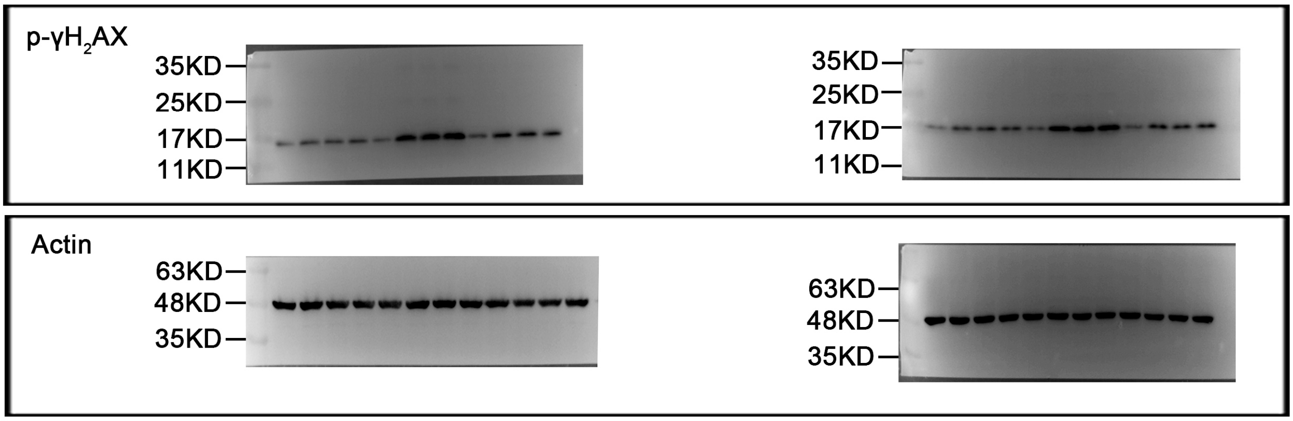
p-γH2AX
35KD
25KD
17KD
11KD
35KD
25KD
17KD
11KD
Actin
63KD
48KD
35KD
63KD
48KD
35KD

Fig. S12A

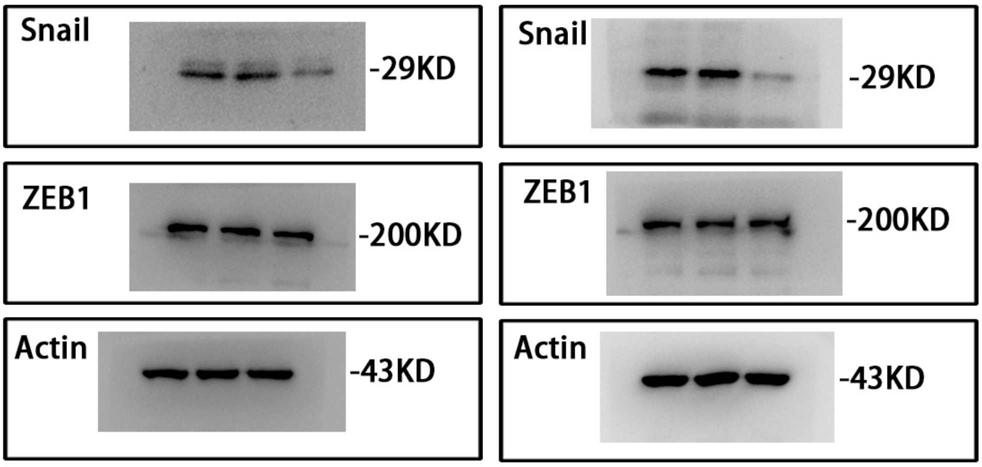

Fig. S12B

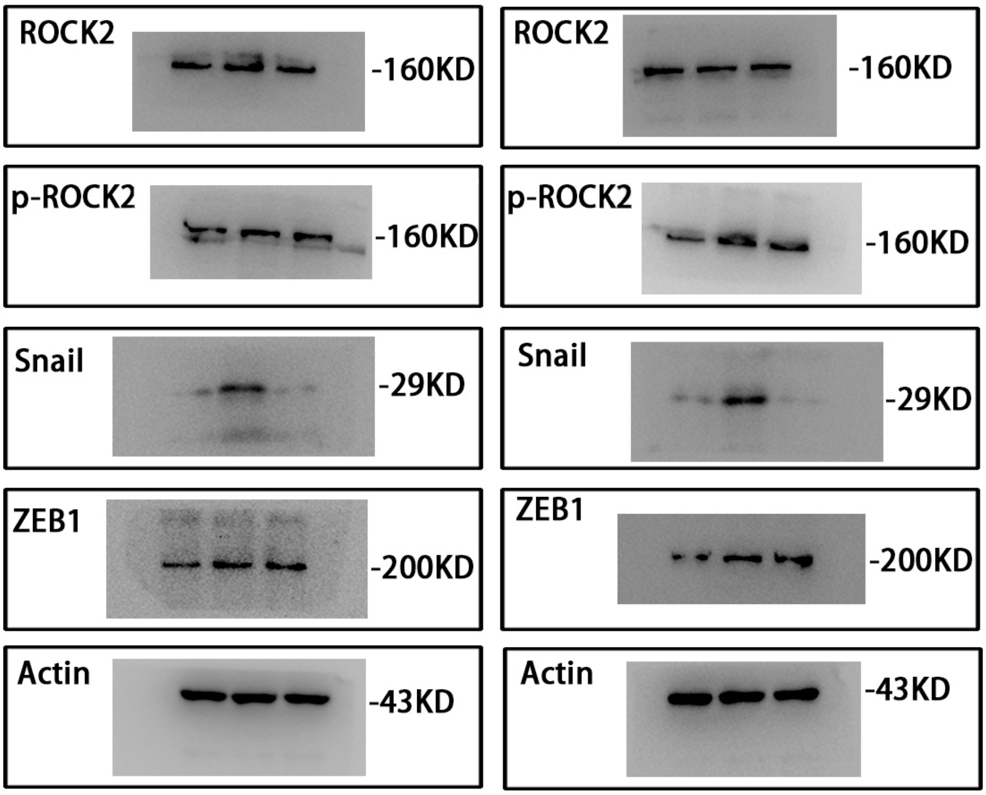

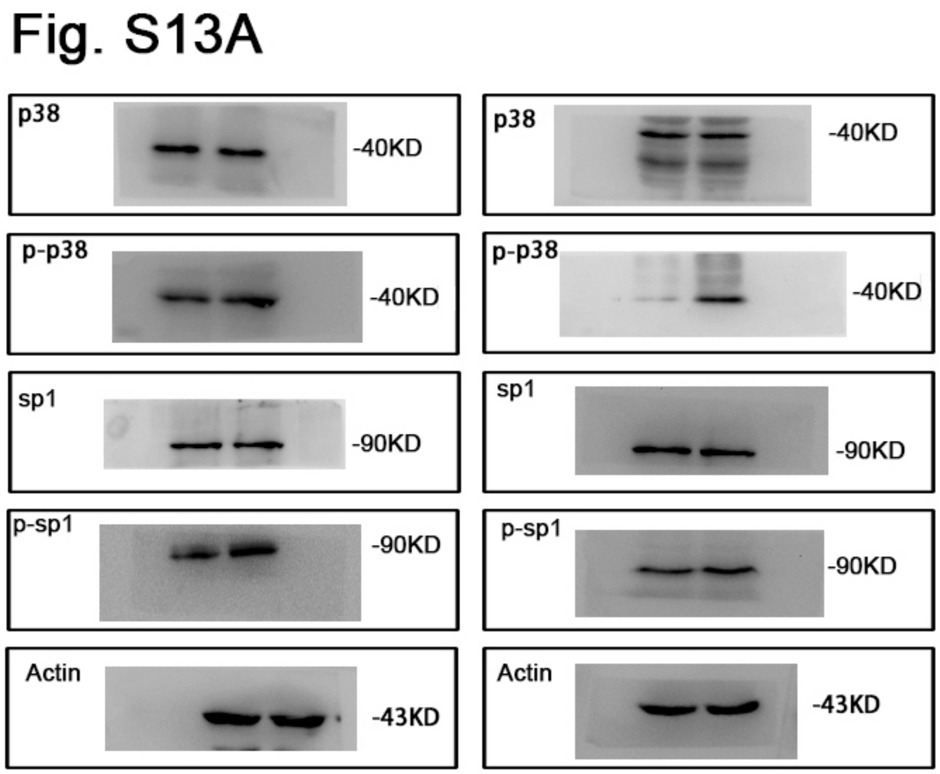
Fig. S13A
p38
-40KD
p38
-40KD
p-p38
-40KD
p-p38
-40KD
sp1
-90KD
sp1
-90KD
p-sp1
-90KD
p-sp1
-90KD
Actin
-43KD
Actin
-43KD

Fig. S13B

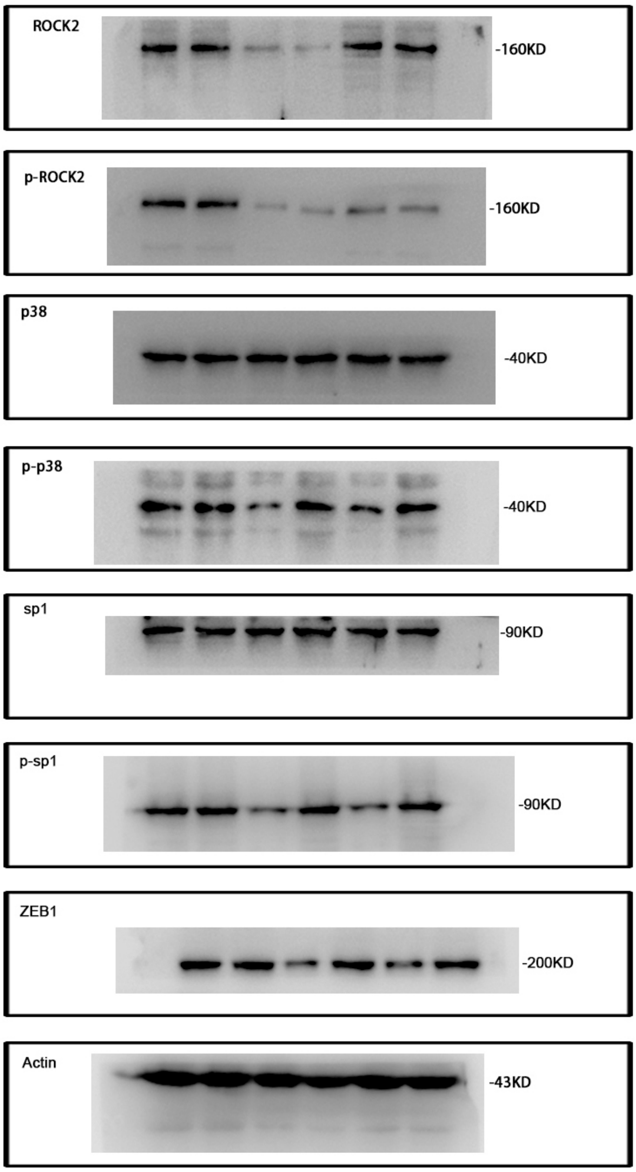
ROCK2
-160KD
p-ROCK2
-160KD
p38
-40KD
p-p38
-40KD
sp1
-90KD
p-sp1
-90KD
ZEB1
-200KD
Actin
-43KD

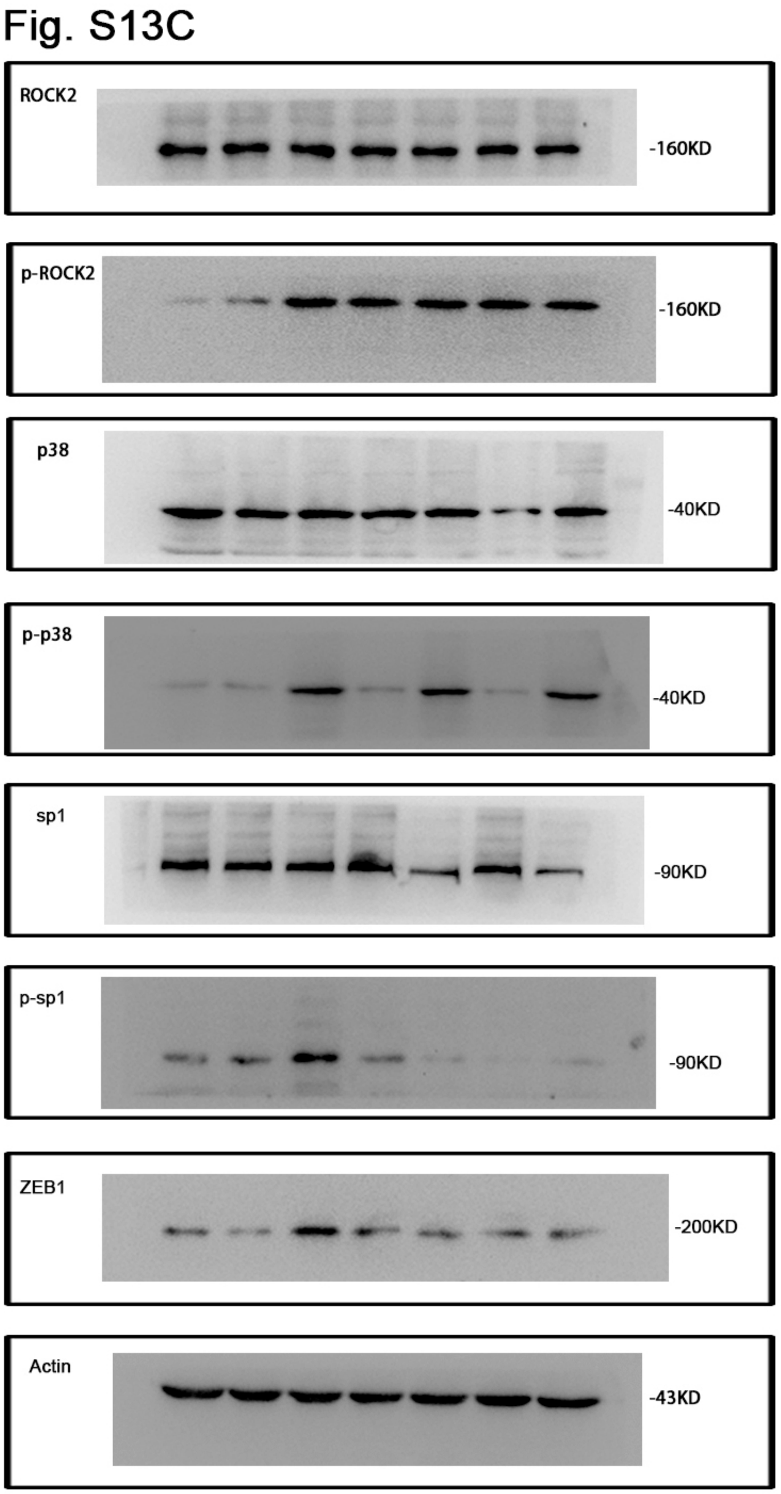
Fig. S13C
ROCK2
-160KD
p-ROCK2
-160KD
p38
-40KD
p-p38
-40KD
sp1
-90KD
p-sp1
-90KD
ZEB1
-200KD
Actin
-43KD

Fig. S13D

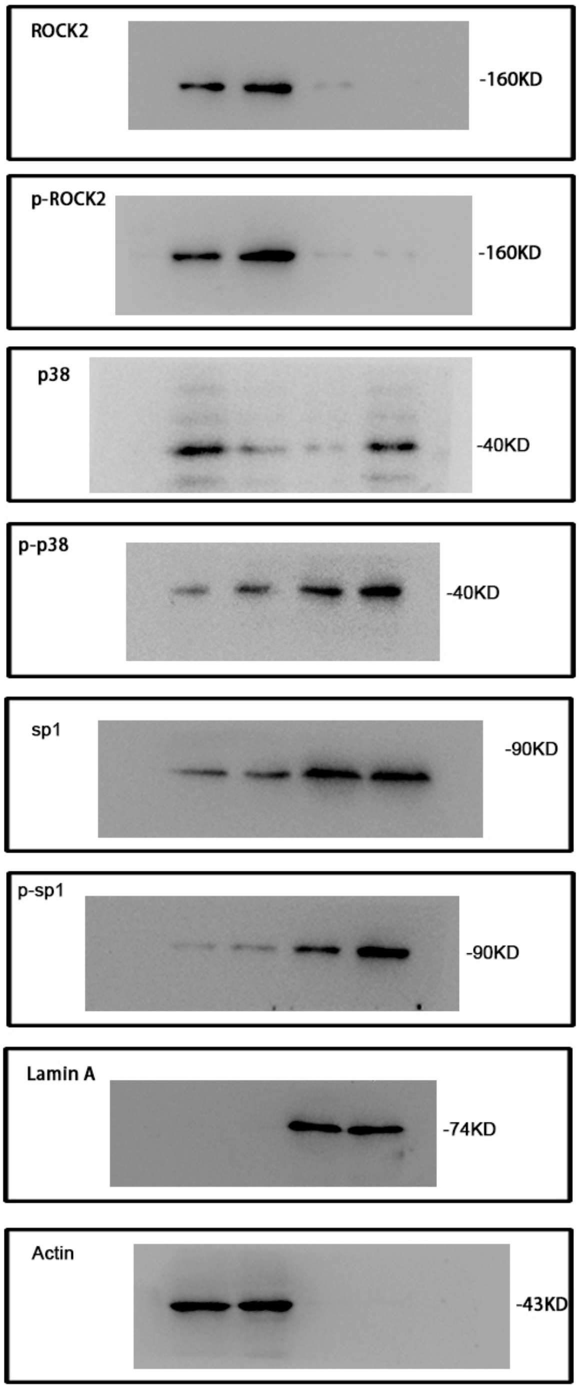
ROCK2
-160KD
p-ROCK2
-160KD
p38
-40KD
p-p38
-40KD
sp1
-90KD
p-sp1
-90KD
Lamin A
-74KD
Actin
-43KD

Fig. S13E

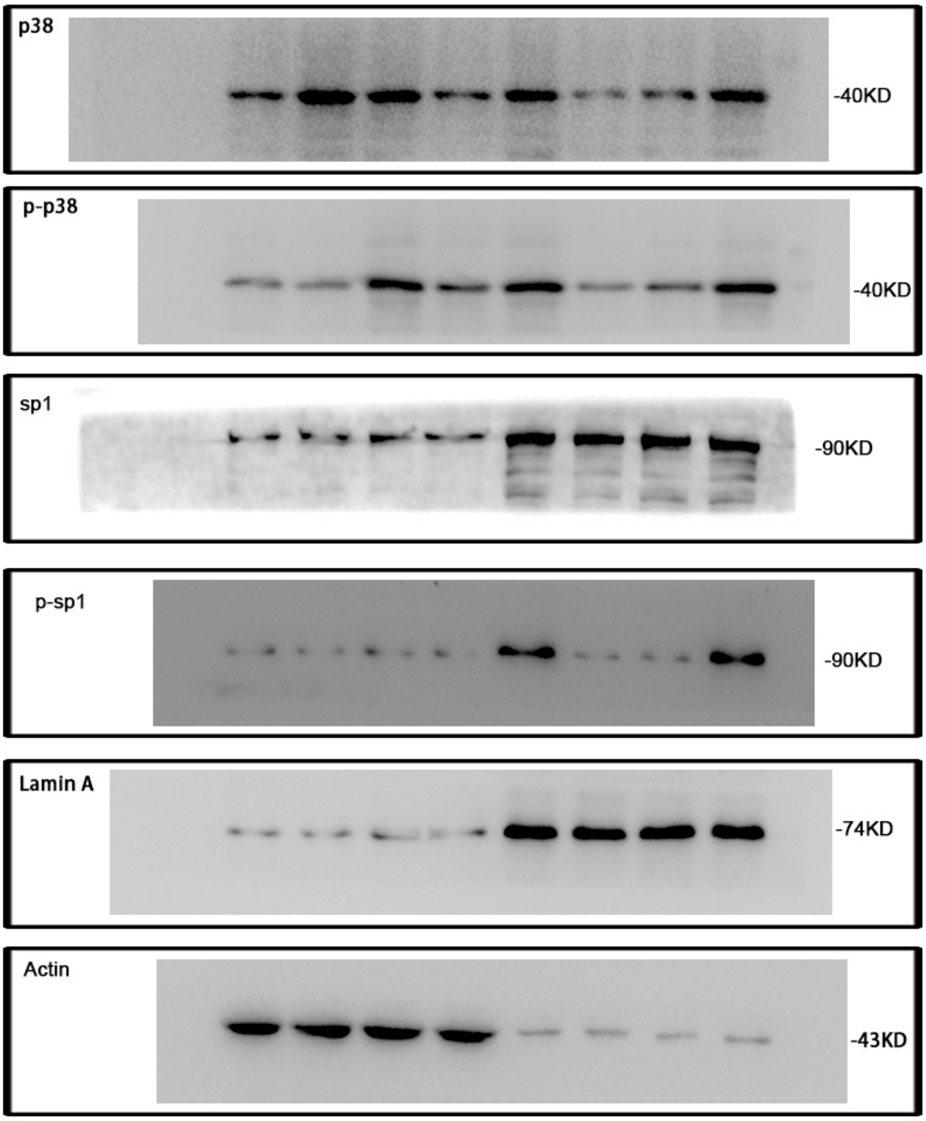

Fig. S15

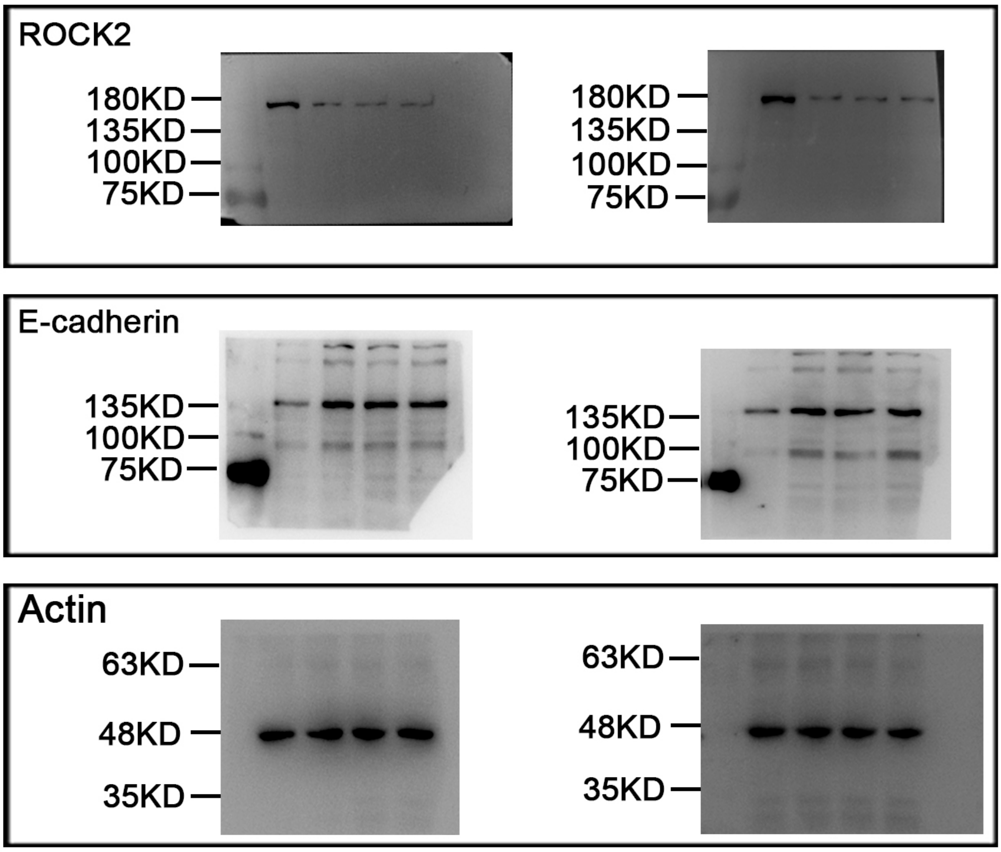

© 2019 by the authors. Submitted for possible open access publication under the terms and conditions of the Creative Commons Attribution (CC BY) license (http://creativecommons.org/licenses/by/4.0/).
